# Psychosocial implications, acceptability and ethics of screening for paediatric type 1 diabetes: a systematic review and mixed methods evidence synthesis

**DOI:** 10.1007/s00125-026-06717-2

**Published:** 2026-05-01

**Authors:** Lauren M. Quinn, David Stanley, Francesca Milano, Gaia Perego, Matthew J. Randell, Francesca Gatti, Antonio Catarinella, Lavinia B. Dettori, Rocco Sheldon, Muhammad Abid, Anette-Gabriele Ziegler, Ezio Bonifacio, Chantal Mathieu, Mark Peakman, Jessica L. Dunne, Carmen Hurtado del Pozo, Jurgen Vercauteren, Lut Overbergh, Emanuele Bosi, Valentina E. Di Mattei, Katharine Barnard, Karin Lange, Renuka P. Dias, Ian Litchfield, Sheila M. Greenfield, Felicity K. Boardman, Olga Boiko, Parth Narendran, EDENT1FI consortium, P. Gillard, K. Casteels, B. van der Schueren, P. Achenbach, F. Haupt, C. Winkler, S. Hummel, F. Reschke, O. Kordonouri, T. von dem Berge, T. Pieber, J. Mader, S. Moitzi, S. Del Prato, B. Torbeyns, Z. Sumnik, O. Cinek, V. Neuman, B. Berka, F. Pociot, B. Petersen, J. Antvorskov, J. H. Klaebel, J. E. Laiho, H. Hyöty, R. Berner, A. Hommel, A. Loff, G. Gemulla, N. Zubizarreta, L. Piemonti, V. Lampasona, F. Dotta, G. Sebastiani, A. Szypowska, K. Karczewski, P. Jarosz-Chobot, J. F. Raposo, R. Ribeiro, R Coelho, E. Niemoeller, M. Baccara-Dinet, J. Van Rampelbergh, R. Bergholdt, K. Fogh, O. Cohen, M. I. Buompensiere, E. Latres, G. Agiostratidou, A. Koralova, J. Jackson, D. Darrock, H. Tewson, S. Nagallas, J. Hedrick, K. Collins, T. Tree, C. Dayan, K. Hood, J. Townson, R. Playle, R. Besser, S. Chen, Marie Amoroso, D. Agardh

**Affiliations:** 1 Institute of Immunology and Immunotherapy, College of Medicine and Health, University of Birmingham, Birmingham, UK; 2 College of Medicine and Health, University of Birmingham, Birmingham, UK; 3 Department of Psychology, University of Milan-Bicocca, Milan, Italy; 4 School of Psychology, Vita Salute San Raffaele University, Milan, Italy; 5 Clinical and Health Psychology Unit, IRCCS San Raffaele Hospital, Milan, Italy; 6 Applied Health Directorate, Warwick Medical School, University of Warwick, Warwick, UK; 7 Institute of Diabetes Research, Helmholtz Munich, and Forschergruppe Diabetes, Klinikum rechts der Isar, Technical University Munich, Munich, Germany; 8 Center for Regenerative Therapies Dresden (CRTD), Faculty of Medicine, Technische Universität Dresden, Dresden, Germany; 9 Laboratory of Clinical and Experimental Endocrinology, Katholieke Universiteit Leuven (KULEUVEN), Leuven, Belgium; 10 Immunology & Inflammation Research Therapeutic Area, Sanofi, MA USA; 11 Sanofi US, Bridgewater, NJ USA; 12 Research Department, Breakthrough T1D, New York, NY USA; 13 Department of Chronic Diseases and Metabolism, KU Leuven, Leuven, Belgium; 14 Diabetes Research Institute, IRCCS San Raffaele Hospital and Vita Salute San Raffaele University, Milan, Italy; 15 Southern Health NHS Foundation Trust, Southampton, UK; 16 Department of Medical Psychology, Hannover Medical School, Hannover, Germany; 17 Department of Applied Health Sciences, College of Medicine and Health, University of Birmingham, Birmingham, UK

**Keywords:** Children, Ethics, Psychosocial, Screening, Systematic review, Type 1 diabetes

## Abstract

**Aims/hypothesis:**

Paediatric population screening for type 1 diabetes is emerging internationally. It is critically important to understand the acceptability of screening to inform these initiatives. In this systematic review, we aimed to assess the psychosocial impact, acceptability and ethics of screening for paediatric type 1 diabetes.

**Methods:**

We searched MEDLINE, EMBASE, APA PsycInfo, ASSIA, CINAHL, Web of Science, Scopus, and included quantitative, mixed methods and qualitative articles until 25 November 2025. We assessed the emotional, cognitive and behavioural implications, acceptability or ethics of type 1 diabetes early detection for parents and/or children. We used the mixed methods appraisal tool and critical appraisal skills checklists for quality assessment. We performed a mixed methods evidence synthesis, identifying key themes from qualitative data and merging with quantitative data to generate meta-inferences.

**Results:**

Seventy articles (12 qualitative, 57 quantitative and one mixed methods) involving 62,244 parents and 6363 children aged <18 years were included. Seven articles (10.0%) met all quality criteria (high quality), 43 (61.4%) met 60–80% of criteria (moderate quality) and 20 (28.6%) met <50% of criteria (low quality). We generated five themes and 19 sub-themes. Identification of early-stage type 1 diabetes generated anxiety, which waned over time but could recur. Overall, parents who opted into an early detection research programme valued knowing their child’s risk and perceived benefits to outweigh harms, although paediatric blood sampling was considered challenging. Research ethics of screening centred on joint decision making according to the child’s age, right to results disclosure and importance of data integrity. We synthesised a large pool of heterogenous studies, reflecting how understanding of early disease has evolved, but likely influencing the acceptability of screening.

**Conclusions/interpretation:**

This, the most comprehensive review of the literature to date, demonstrates that despite the emotional, cognitive and behavioural implications, thus far, screening and early detection of paediatric type 1 diabetes appears to be acceptable to parents/guardians who take part but critical evidence gaps remain.

**Trial registration:**

PROSPERO registration no. CRD42024566937

**Funding:**

EDENT1FI (grant no. 101132379)

**Graphical Abstract:**

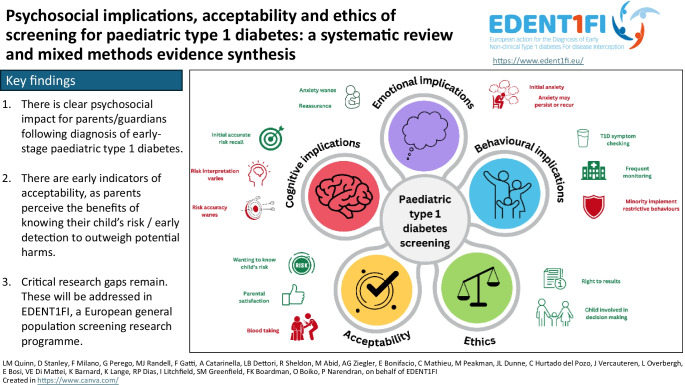

**Supplementary Information:**

The online version contains peer-reviewed but unedited supplementary material available at 10.1007/s00125-026-06717-2.


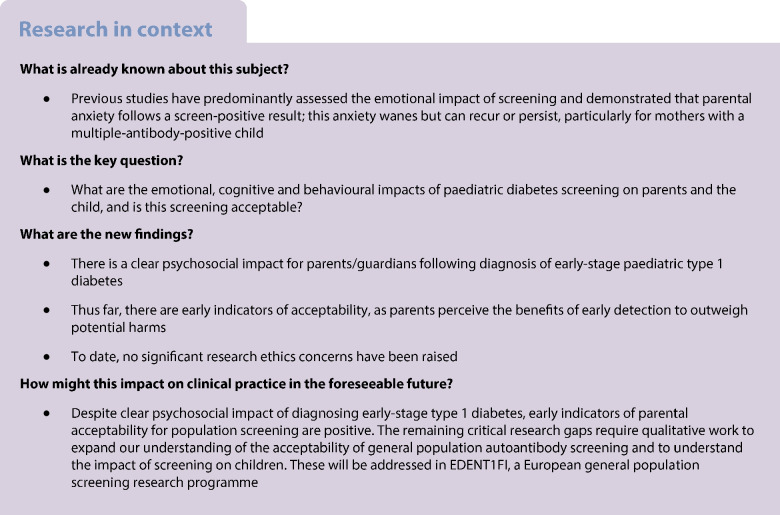


## Introduction

Type 1 diabetes is an autoimmune disease caused by destruction of insulin-producing beta cells, leading to lifelong insulin dependence [1]. The key approaches to identifying early-stage type 1 diabetes include genetic and islet-specific autoantibody (Aab) testing [2]. Genetic testing identifies high-risk individuals who could benefit from serial Aab monitoring. Aab testing identifies children with early-stage disease months to decades prior to insulin requirement (stage 3) [3].

Clinical benefits of early detection include an up to fivefold reduction in diabetic ketoacidosis (DKA) [4, 5], a milder clinical phenotype at presentation [6], and access to trials and therapies to delay progression [7]. However, psychosocial and ethical issues surround the identification of presymptomatic paediatric conditions [8, 9]. Reviews show that parental anxiety, which follows high-risk notification for type 1 diabetes, generally wanes but so does accurate risk perception [10–12]. There has not been a systematic review in this area, nor formal synthesis of the acceptability, ethics or qualitative data around the impacts of screening. New studies of general population Aab screening [4, 13] are generating high-quality data regarding psychosocial implications and as our understanding has evolved [3], progression rates conveyed to families have improved from 1 in 16 likelihood [14] to current near lifetime certainty of type 1 diabetes [4]. These studies are underway worldwide, with significantly more programmes planned and Italy becoming the first country to mandate population screening [15]. One of the largest of these research initiatives is the ongoing European EDENT1FI programme (grant no. 101132379, https://www.edent1fi.eu/), an encompassing study investigating the feasibility, acceptability and long-term impacts of general population Aab screening via quantitative and qualitative methods. Synthesising evidence on screening impacts from the parent and child’s perspectives is crucial to inform these initiatives.

This is the first systematic review in this field and collates three decades of evidence surrounding the emotional, cognitive and behavioural impacts of early detection of paediatric type 1 diabetes, including overall acceptability and consideration of research ethics.

## Methods

The protocol was registered with PROSPERO (https://www.crd.york.ac.uk/PROSPERO/view/CRD42024566937) and follows Preferred Reporting Items for Systematic Reviews and Meta-Analyses (PRISMA) reporting guidelines. There were no deviations from the PROSPERO registered protocol.

### Search strategy

We searched seven databases (MEDLINE [OVID], EMBASE [OVID], Web of Science, American Psychological Association [APA] PsycInfo, Applied Social Sciences Index and Abstracts [ASSIA], Cumulative Index to Nursing and Allied Health Literature [CINAHL] and Scopus) until 25 November 2025. Search terms were refined following iterative scoping searches. We used a variety of medical subject headings (MeSH) and synonyms to identify studies that screened children for type 1 diabetes (population), in the context of a targeted (family members) or population screening programme or birth cohort study (interventions) and assessed the experience, implications and/or acceptability of screening (outcomes). There were no restrictions by language, country or publication year. The primary search strategy is included in electronic supplementary material (ESM Methods). The secondary search strategy comprised forward and backward searching of reference lists of included studies and relevant reviews. To identify grey literature, we searched the following open-access repositories: Health Management Information Consortium (HMIC); Open Access Infrastructure for Research in Europe (OpenAIRE); and the Social Science Research Network (SSRN).

### Article selection

Citations were imported into Covidence and duplicates removed. Titles and abstracts were screened independently (LQ/DS) and disagreements resolved by discussion with a moderator (OB). Reviewers applied pre-specified criteria to determine eligibility (ESM Table 1). Two reviewers independently extracted data (LQ/DS) into Microsoft Excel.

Inclusion criteria were defined by population, intervention, comparator and outcomes (PICO). We included studies that tested children (aged <18 years) for type 1 diabetes via genetic, Aab or combined approaches. Study designs included prospective cohorts, observational, non-randomised and cross-sectional, adopting quantitative, qualitative or mixed methodologies. We assessed parent and/or children’s emotional, cognitive, behavioural implications, acceptability and research ethics considerations. Comparators comprised unscreened or usual care cohorts.

### Quality assessment

We used the Mixed Methods Appraisal Tool (MMAT) corroborated by the Critical Appraisal Skills Programme checklists (CASP) for quality assessment [16, 17]. Two reviewers performed quality assessments independently (LQ/DS) and a third reviewer moderated (OB). Quality was designated as high (>90% criteria met), moderate (50–90%) or low (<50%). Findings from high and moderate quality studies were prioritised. The MMAT question about appropriateness of the psychosocial assessment tool used was modified to give weighting to validated instruments.

### Thematic synthesis and mixed methods convergence

Study design and outcome heterogeneity precluded meta-analysis. We performed inductive thematic synthesis using the three-stage process described by Thomas and Harden [18]. Stage 1 involved iterative, line-by-line coding in Microsoft Excel of the primary qualitative results; this included reading and re-reading articles to identify similarities and differences and translate findings. Here, we found the available evidence fitted into one of three categories: psychosocial impact; acceptability; and research ethics. We (LQ/DS) then sub-classified the data items into five distinct outcomes: emotional impact; cognitive impact; behavioural impact; acceptability; and ethics. Acceptability is a multi-faceted construct and we based its definition on Sekhon et al’s theoretical framework of acceptability (TFA) [19]. Aspects of the TFA identified in the included articles were affective attitude, satisfaction, burden, value, balance of benefit and harm, and reasons for continuation or discontinuation. Constructs of the TFA that were not identified included perceived effectiveness, intervention coherence, opportunity costs and self-efficacy. Data pertaining to ethics/ethicality of screening centred on research ethics (i.e. consent, confidentiality and results disclosure) as opposed to bioethical models of whether screening is ethical or not.

Stage 2 involved development of descriptive themes, which remained close to the primary articles’ findings. All relevant extracts for each theme and sub-theme were collated [20]. Themes were checked for distinctiveness and coherence, and compared with the original data extractions.

Stage 3 involved generation of analytical themes to go beyond the primary data (third-order evaluation) and provide novel interpretation of findings to identify potential new constructs. The themes and sub-themes were tabulated with exemplar quotes (LQ; verified by OB/DS/RPD/FB). To integrate the qualitative and quantitative results, we performed an explanatory sequential design, first analysing the qualitative data to identify themes and sub-themes [21]. Then, we applied a statistics-by-theme approach to the quantitative data, highlighting aspects of confirmation, expansion or discordance. This involved highlighting whether findings from the qualitative and quantitative data were consistent and reached the same conclusion (confirmation), provided new insights and enabled greater understanding beyond the original data (expansion) or whether data were conflicting indicating further research is needed (discordance) [22]. This generated an overall direction of effect and meta-inferences with conclusions drawn from the combined data [23]. Supporting evidence was graded according to adequacy, fit and quality. Finally, we report on characteristics identified from the quantitative data (i.e. demographic and clinical factors associated with psychosocial impact).

## Results

**Fig. 1:**
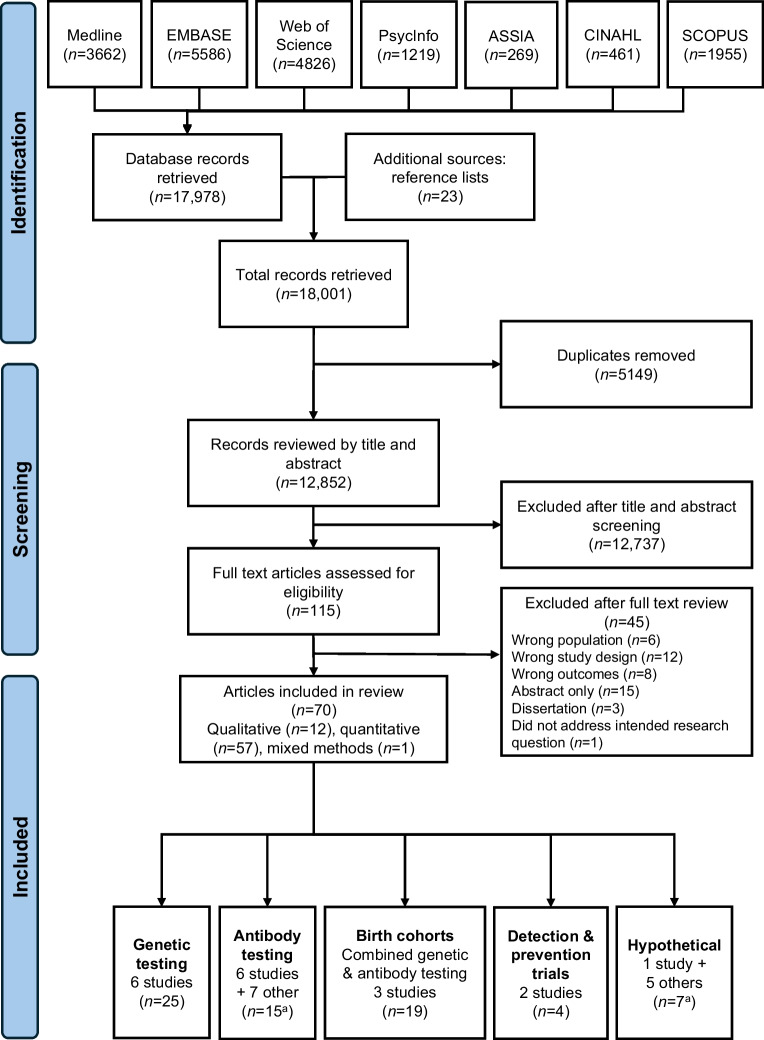
PRISMA 2020 flow diagram. ^a^Reference 62 had data pertaining to ‘Antibody testing’ and ‘Hypothetical' but is grouped in the ‘Antibody Testing’ category here

The database searches identified 17,978 potentially relevant articles plus 23 articles from reference lists. Of these, 70 met the pre-specified inclusion criteria (Table 1 and ESM Tables 2, 3), comprising 12 qualitative (17.1%), one mixed methods (1.4%) and 57 quantitative articles (81.4%) (Fig. 1). All articles were peer-reviewed and the studies conducted in high-income countries (one Australia, one Belgium, two Canada, two Finland, four Germany, one Israel, one Italy, one the Netherlands, four New Zealand, one Norway, 15 Sweden, six UK, 14 USA, 16 multi-centre Europe and USA, one multi-centre Europe, USA, Canada, Australia, New Zealand). No grey literature was identified.

**Table 1:** Summary of included studies

Approach to detection	No. of articles	Data	Outcomes assessed
Genetic testing (genetic)			
ABIS birth cohort	13	Quantitative/qualitative	Emotion, cognition, acceptability, ethics
DAISY birth cohort	1	Quantitative	Emotion
MOBA/MIDIA birth cohort	1	Quantitative	Emotion
PANDA	5	Quantitative	Emotion, cognition, behaviour
Freder1k	1	Quantitative	Emotion
KEA	4	Quantitative/qualitative	Emotion
Antibody population screening (Aab, general population)			
ASK	1	Quantitative	Emotion, cognition, acceptability
Fr1da	2	Quantitative	Emotion, cognition, acceptability
Type1Screen	1	Quantitative	Emotion, cognition, acceptability
UNISCREEN	1	Quantitative	Emotion, cognition, acceptability
Antibody testing (Aab)			
T1Early	1	Qualitative	Emotion, cognition, behaviour, acceptability
Trialnet (FHx)	2	Quantitative	Emotion, cognition, behaviour, acceptability
Other	7^a^	Quantitative	Emotion, cognition, behaviour, acceptability
Birth cohorts (combined genetic and antibody surveillance)			
TEDDY	16	Quantitative	Emotion, cognition, behaviour, acceptability
DIPIS	2	Quantitative	Emotion
BABYDIAB (FHx)	1	Quantitative	Emotion
Detection and prevention trial (genetic testing followed by intervention)			
DIPP	2	Quantitative	Emotion, acceptability
GPPAD	2	Quantitative	Emotion, acceptability
Hypothetical			
Other	5^a^	Qualitative, mixed methods	Acceptability, ethics
ELSA	3	Qualitative	Acceptability, ethics

Outline of the screening study groups: genetic (*n*=25); antibody (Aab) (*n*=15); birth cohorts (*n*=19); detection and prevention trial (*n*=4); hypothetical (*n*=8)

^a^Total *n*=71 because one study included data supporting Aab testing and another dataset supporting hypothetical views

FHx, participants having a family member with type 1 diabetes

Fifty-eight (82.9%) articles assessed parents’ views, five (7.1%) children’s views and seven (10.0%) both. In total, the articles included 62,244 parents and 6,363 children. The quantitative and mixed methods studies involved 61,902 parents (highest number reported for the uncontrolled cohorts/time series) and 6314 children aged <18 years, from 1997–2025. The 12 qualitative studies comprised 342 parents and 49 children aged 5–12 years.

Of the quantitative articles, there were 19 uncontrolled prospective cohort studies (>13 months), 18 uncontrolled time series (≤13 months) and 20 cross-sectional studies. The median length of follow-up was 36 months for cohort studies and 6 months for time series. The mixed methods study included interviews (unpublished) informing a discrete choice experimental (DCE) survey. Of the qualitative articles, *n*=7 performed semi-structured interviews, *n*=2 workshops, *n*=1 focus group, *n*=1 thematic analysis from mailbox responses and *n*=1 secondary analysis.

Of the 70 included articles, 62 (88.6%) explored implications or acceptability for screened/tested cohorts, seven (10.0%) explored hypothetical views (i.e. unscreened) and one explored both (1.4%). Type 1 diabetes screening/testing approaches included genetic (*n*=25 articles), Aab (*n*=15), combined detection and surveillance (*n*=19 articles) and detection with a prevention trial (*n*=4 articles). Included articles were drawn from 17 named screening studies (Table 1 and ESM Table 2). ABIS, DAISY, Freder1k, KEA, MOBA/MIDIA and PANDA offered genetic testing followed by variable Aab surveillance (genetic). TEDDY, DIPIS and BABYDIAB are prospective birth cohort studies investigating natural history and offering Aab surveillance (birth cohort). DIPP and GPPAD are combined genetic screening and primary prevention studies. Fr1da, ASK, Type1Screen, UNISCREEN and T1Early offer general population Aab screening (Aab, general population), while Trialnet offered Aab testing to individuals with a family history of type 1 diabetes (FHx).

Evaluation of article quality using the MMAT showed that seven articles (10.00%) met all quality criteria (high quality), 43 (61.43%) met 60–80% of criteria (moderate quality) and 20 (28.57%) met <50% of criteria (low quality). CASP and MMAT scores were compared (ESM Table 4).

### Themes and sub-themes

**Fig. 2:**
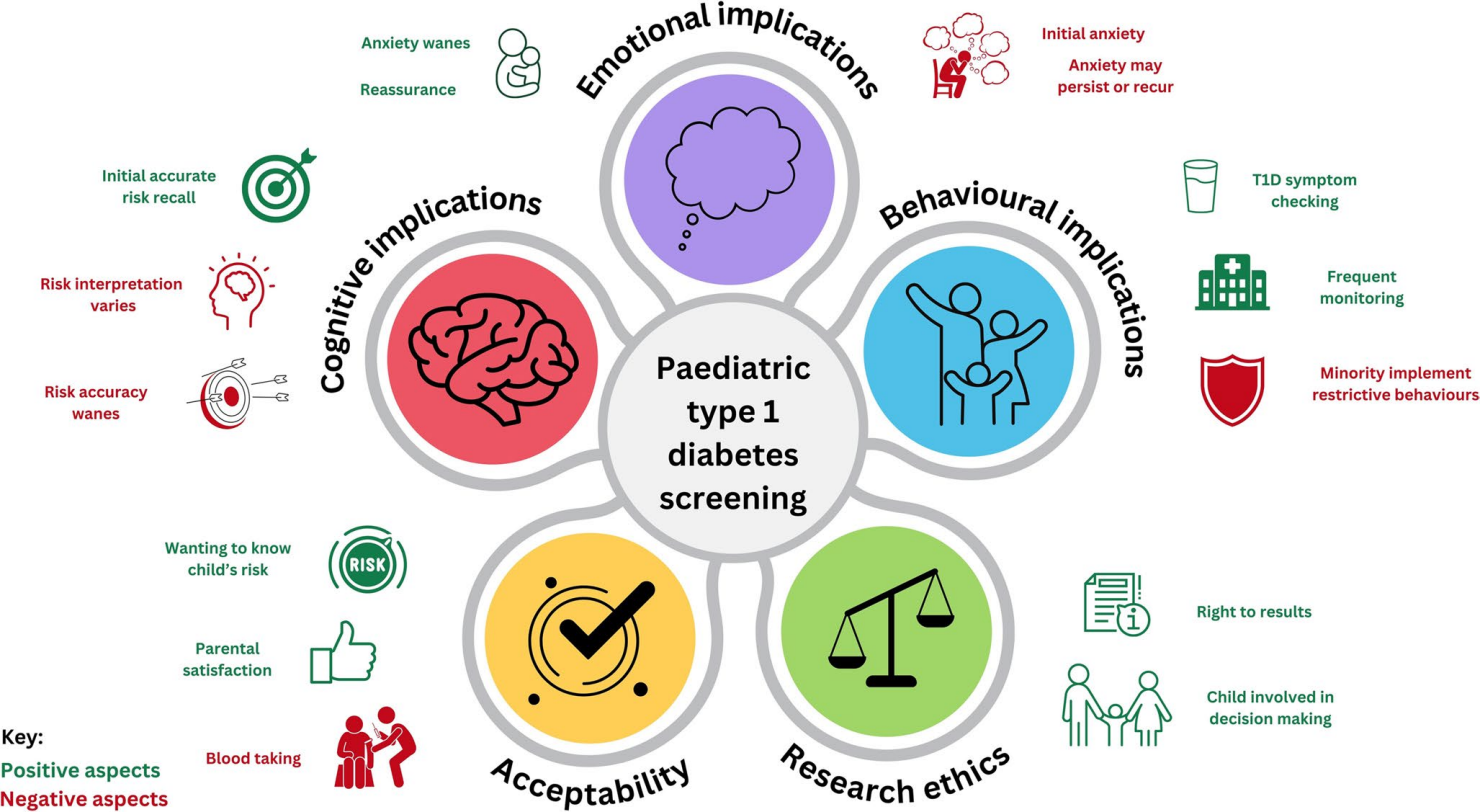
Summary of findings from the mixed methods evidence synthesis. Positive and negative aspects of paediatric type 1 diabetes screening relating to implications, acceptability and research ethics considerations are highlighted in green and red, respectively. Created in https://www.canva.com/. Canva Pro license agreement: Under the terms of this agreement, Canva grants a perpetual, non-exclusive, non-transferable, worldwide license to use Pro Content in a single Canva Design (Fig. 2), for the Permitted Use for online or electronic publications (https://www.canva.com/policies/content-license-agreement/). You may not copy, download or distribute the Pro Content (Fig. 2) as a standalone item

The mixed methods evidence synthesis identified five themes, including emotional, cognitive and behavioural implications, acceptability and research ethics, plus 19 sub-themes. Table 2 provides the joint display with qualitative and quantitative data summaries, evidence grading and meta-inferences (see also Fig. 2 and ESM Table 5) [22].

**Table 2:** Main results

Theme and sub-theme	Exemplar quote^a^	Meta-inferences (combining qualitative and quantitative data)	Concordance between qualitative and quantitative data (confirmation, expansion or discordance)	Overall direction of effect	Quality of supporting articles	Adequacy of supporting articles, no. of articles (*n* qualitative : *n* quantitative)	Supporting references
Emotional implications							
Initial anxiety	*‘...when he was diagnosed it did send me into a bit of a panic.’* (P12), Kerruish 2016 [24]	Immediate reaction to high-risk results notification is increased anxiety (and depression) but the initial shock is usually short-lived, lasting weeks	Expansion	Negative	Low-high	21 (2:19)	[4, 13, 14, 24–41]
Anxiety can wane	*‘At the time we were a wee bit shocked by it, but now we just carry on and it hasn’t been an issue to us or anything,’* (P7), Kerruish 2016 [24]	Parental anxiety (and depression) and children's anxiety waned in the months following results notification Despite initial anxiety, parents felt reassurance from screening participation	Confirmation	Positive	Moderate-high	7 (1:6)	[24, 27–29, 33, 42, 43]
Anxiety may persist or recur	*‘It doesn’t get thought about a lot, it doesn’t, it is when I get the letter that says X is due for her blood* test' (P6), Kerruish 2011 ‘*oh my God he’s got it moment*’ (P5), Kerruish 2011 [25]	Parents experience occasional lingering concerns, particularly around monitoring visits or emergence of T1D symptoms	Confirmation	Negative	Moderate-high	5 (2:3)	[13, 24, 25, 31, 33]
Characteristics associated with anxiety		Quantitative studies revealed demographic subgroups who experience heightened anxiety following high-risk notification, including minority ethnic groups, parents with lower educational level, single parents and mothers with pre-morbid mental health/anxiety	Quantitative only	Negative	Low-high	13 (1:12)	[13, 14, 25, 29, 31–34, 36, 37, 40, 42, 44]
Cognitive implications							
Initial accurate risk perception	*‘To me it just meant that because he had some kind of indicator, genetic indicator, that he was more susceptible perhaps to diabetes.’* (P12), Kerruish 2016 [24]	Most parents recalled the ‘gist’ of the risk information (numerically or descriptively) appropriately The strongest predictor of accurate or high-risk perception was parents of children with FHx	Confirmation	Positive	Moderate-high	5 (1:4)	[13, 24, 35, 42, 45]
Risk interpretation varied	*‘Basically it is still only a 10% chance or even less that she will get diabetes so we don’t really need to worry,’* (P4), Kerruish 2011 [25] ‘*I don’t like his odds; it would be much better if he was in the one in 300 chance as opposed to the one in 16. Someone is going to get it aren’t they?*’ (P5), Kerruish 2011 [25]	Risk interpretation was determined by the value placed on the result Despite parents recalling broadly similar degrees of risk, the parent's interpretation/meaning and the perceived likelihood of their child developing T1D differed between parents and intra-individually over time Making sense of risk was a dynamic process that involved the parent locating their child's diabetes risk status on a map of relative risks/potential illnesses or situations they considered as more serious or more likely	Expansion	Negative	Moderate-high	8 (2:6)	[14, 24, 25, 27, 28, 32, 34, 35]
Accurate risk perception declined	*‘But then he is perfectly fine and I must admit, as he has got older I have got less concerned,’* (P8), Kerruish 2016 [24]	Risk accuracy wanes leading to risk underestimation Increased time since results notification meant the parents thought less about the results (*'forgot about it',* Kerruish 2016 [24]) and were reassured by child's good health as they got older Parents felt the risk status should be accepted and gotten used to	Confirmation	Negative	Moderate-high	7 (2:5)	[24, 25, 33, 35, 42, 45, 47]
Characteristics associated with accurate risk perception		Quantitative studies revealed demographic subgroups who experience increased risk accuracy, including FHx parents, high-risk classification and mothers with higher anxiety, whereas under-represented groups had lower risk accuracy	Quantitative only	Mixed	Low-high	14 (0:14)	[13, 29, 31, 33, 34, 36, 37, 42, 44–49]
Behavioural implications							
Behavioural strategies	*‘It was common for parents to stress that they would consider the diagnosis if their child became unwell with symptoms suggestive of T1D.*' Kerruish 2016 [24]	The majority of parents adopted behavioural strategies with the aim of preventing/reducing harm from future T1D Parents who minimised the value of the high-risk results less-frequently engaged in monitoring behaviours; these parents described not having time to worry about type 1 diabetes every day On the other hand, perceived high-risk and anxiety predicted maternal efforts to prevent T1D (Baughcum 2005 [50]) Parents who attached most value to the child's risk status described frequent monitoring behaviours, including contacting healthcare providers for advice (Kerruish 2011 [25])	Confirmation	Positive	Moderate-high	10 (2:8)	[24–26, 28, 32, 34, 44, 48–50]
Monitoring behaviours	*‘ said to him, “what about this drinking because he drinks a lot?” And he said, “you are worried about diabetes aren’t you?”,’* (P4), Kerruish 2011 [25]	Health surveillance/monitoring was the most frequent 'preventative' behaviour, including participation in a follow-up study e.g. TEDDY, symptom monitoring and glucose monitoring These behaviours occurred more frequently following high-risk identification Both symptom monitoring and glucose monitoring occurred more frequently in those with FHx	Expansion	Positive	Moderate-high	6 (2:4)	[24, 25, 44, 49–51]
Lifestyle changes	*‘I honestly don’t know if this is a result of the information I was given or something I would have done anyway...but we don’t eat anything with any added preservatives...or colours. We make as much of our stuff as what we can,’* (P10), Kerruish 2016 [24]	High-risk status was an incentive for the family (parent and child) to lead as healthy a lifestyle as possible In practice, most families said they had not made any lifestyle changes or it was difficult to say if healthy lifestyle changes were due to the high-risk result Lifestyle changes included healthy eating (low energy, low sugar) and increased physical activity and if promoted, appeared to apply to the whole family	Expansion	Positive	Moderate-high	6 (2:4)	[24, 25, 28, 44, 48, 50]
Restrictive behaviours	*‘We were giving them kind of a vitamin D...supplement in the winter. And just kind of encouraging them to go outside...I think I read something which suggested that vitamin D was to protect them from those kinds of diseases,’* (P15), Kerruish 2016 [24]	Parents rarely adopted (1–9%) overly restrictive/protective behaviours Overly protective behaviours were employed with intent to reduce child's risk of T1D, including: (1) deciding not to have further children; (2) prolonged breast feeding; (3) vitamin supplementation; (4) illness prevention; (5) stress reduction; and (6) changes to future planning (profession)	Confirmation	Negative	Moderate-high	7 (2:5)	[24–26, 34, 41, 49, 50]
Characteristics associated with behavioural implications		Significant factors associated with behavioural modifications at 6 months included older maternal age, higher maternal education, only child, FHx, higher anxiety or postpartum depression, accurate risk perception and belief that risk was modifiable The strongest factors associated with monitoring behaviours included FHx parents and accurate risk perception compared to parents who underestimated their child’s risk	Quantitative only	Negative	Moderate	1 (0:1)	[44]
Acceptability							
Motivation for early detection	*‘Well, there’s definite benefits. I mean it’s, anything that’s, you know, looked at early if there is that sort of thing going to happen or if it is starting to develop well then there’s, there’s definite benefits to that,’* (P3), Kerruish 2016 [24]	Parents valued opportunities to participate in screening to benefit from time to prepare, follow-up and possibility of prevention strategies T1D or chronic conditions in the family increased relevance of screening Follow-up studies demonstrated majority adherence Hypothetical articles showed parents sought effective treatment for prevention	Expansion	Positive	Low-high	21 (5:16)	[24, 33, 34, 49, 50, 53, 55, 56, 58–60, 62, 64, 65, 69, 75, 77–81]
Reservations about early detection	*‘We said right from the beginning that we did NOT want to be a part of this because we do not want to know. It is not the fear of knowing it is just that, neither one of us wants to know or find out. Why do you want to know what will happen? You can find out if you have cancer genes, and you might need to do that, but why? If I get it, I get it, I can’t protect myself from it. I won’t be happier to know.’* (non-participating mother 4, 23:1), Stolt et al 2002 [77]	Main reservations for screening included blood taking, for fear of hurting the child, and logistical challenges (e.g. travel, lack of time or family factors) Perceived irrelevance drove non-participation in screening Hypothetical studies raised concerns about medical insurance	Expansion	Negative	Low-high	13 (4:9)	[24, 38, 53, 58–61, 63, 64, 66, 69, 75, 77]
Participation experience and preferences	*‘If we didn’t do the* test *we would have been none the wiser. We wouldn’t have known about the symptoms so it was good.’* (P4), Kerruish 2016 [24] *‘Oh the heel prick* test*s were a nightmare. Absolute nightmare with J,’* (P5), Kerruish 2016 [24] Timing – would prefer screening in childhood rather than newborn period, Kerruish 2016 [24]	Parental satisfaction was high and the major downside was blood taking Parents and children preferred less invasive and more convenient screening tests (e.g. capillary blood taking at home) and early detection would be even more acceptable if a prevention treatment was available	Expansion	Positive	Low-high	22 (6:16)	[24, 26–28, 33, 34, 37, 51, 52, 54, 57, 58, 62, 65–71, 75, 77]
Ethics of screening							
Decision making	*‘You decide because you believe it is in the child’s best interests. That is what we are meant to do,’* (participating mother 10, 32:2), Stolt et al 2002 [77] *‘My parents decide if I can. I decide if I want to.’* (child), Swartling et al 2011 [81]	Parents felt sufficient information was provided and felt the child had a right to participate in screening decisions Children preferred joint decision making	Expansion	Positive	Low-high	10 (3:7)	[37, 51, 52, 77, 79–81, 84–86]
Results disclosure	Parental results disclosure: *‘Yes, as the mother of a child who is a part of the study, of course I want to (be informed). It is his right,’* (participating mother 14, 41:2), Stolt et al 2002 [77] Child's results disclosure: *‘Everyone’s different in how they explain things to their kids. We don’t tend to worry them. We've been open with them with everything...it’s a matter of being able to tell them but also reassuring them that this might never happen,’* (P6), Kerruish 2016 [24]	Parents felt they had a right to know their child's risk status and the majority of parents felt the child should be informed	Confirmation	Positive	Low-high	10 (3:7)	[24, 26, 34, 38, 59, 75, 77, 78, 86, 87]
Data integrity	*‘Yes, I believe there should be restrictions. It is positive information, but still sensitive. People will know a lot about him. If he does get ill, a lot of people are going to know about it, they will know what will happen to him and what genes he has. But anyway it is positive,’* (participating mother 15, 25:1), Stolt et al 2002 [77]	Parents felt data confidentiality was important in research participation	Confirmation	Positive	Low-high	8 (1:7)	[59, 77–80, 84–86]

Mixed methods joint display outlining the themes and sub-themes with supporting qualitative, quantitative evidence and meta-inferences [22, 23]

^a^Represents the verbatim quote from the interview participant

#### Theme 1: emotional implications of screening

The systematic review identified emotional implications of screening as having the following sub-themes: initial anxiety; anxiety can wane; anxiety may persist or recur; and characteristics associated with anxiety.

The sub-theme of initial anxiety was explored using 21 articles that assessed the emotional implications of screening: two qualitative [24, 25]; and 19 quantitative [4, 13, 14, 26–41]. Eight articles assessing the emotional impact of genetic testing (KEA, ABIS) reported the following: (1) no statistically significant increase in anxiety compared with baseline (pre-notification); (2) anxiety comparable with normative samples; and/or (3) anxiety below clinical threshold [14, 29, 34, 36–40]. Although up to a quarter of the parents (1–25%) reported worry or anxiety [14, 33, 37, 38], there was no significant difference in anxiety or postpartum depression between high- and low-risk groups [14, 34]. Qualitative articles reported panic and upset following genetic high-risk notification or early-stage type 1 diabetes detection [24, 25].

In four articles assessing the emotional impact of Aab testing, anxiety measured by the state trait or state anxiety inventory (STAI/SAI) after screening participation was significantly increased and/or was above the clinical cut-off [13, 26–28].

For general population Aab screening, following multiple Aab seropositive results notification, Fr1da reported significantly increased median depression scores, measured via the Patient Health Questionnaire 9 (PHQ-9), in mothers of children with presymptomatic type 1 diabetes compared with mothers of Aab-negative control children, with no significant difference observed for fathers. However, the median PHQ-9 score for parents with an Aab-positive or -negative child was below the clinical cut-off for major depressive disorder [4]. In ASK (general population, Aab), parents’ mean anxiety level, assessed via the SAI, was above clinical threshold regardless of Aab status (multiple/single) with no significant difference between groups; 74% experienced SAI above clinical threshold at first follow-up [13].

Most parents (81–96%) participating in the GPPAD intervention study had no symptoms of depression or panic-specific anxiety following high genetic risk notification. However, mothers (20.9–23.3%) displayed higher levels of depressive and anxiety symptoms than fathers (11–15%) [41]. Similarly, in DIPP (detection and prevention trial), high-risk mothers were more likely to display anxiety (55%) than high-risk fathers (37%). Overall, 52–56% said they wanted to do everything to prevent type 1 diabetes [34].

The sub-themes addressing whether anxiety can wane but may persist or recur were explored by evaluating 9 articles that reported the medium-term effects (up to 5 years) of high-risk notification [4, 24, 27–29, 33, 41–43]. Of these articles, seven demonstrated reduction in anxiety and two reported reduction in depressive symptoms [4, 24, 27–29, 33, 41–43]. Following genetic screening, parental anxiety declined over 4 months [29] and at the 5 year follow-up in DIPIS (birth cohort), the majority of parents (67%, *n*=1380) did not report anxiety despite their child being at increased risk of type 1 diabetes; 76% of mothers (*n*=1555) and 86% of fathers said they never or rarely worried about their child developing type 1 diabetes [33]. Up to 12 years after genetic predisposition disclosure in KEA, parental concerns had largely abated, with the test result described as being at the back of their minds [24].

In Fr1da, for mothers and fathers with an Aab-positive child, depression scores had declined significantly by 12 months, remaining below the clinical cut-off throughout [4]. In a US Aab testing study, within 4–6 months of Aab-positive results notification, anxiety levels fell to below the clinical threshold for parents and children [27, 28].

In GPPAD (general population, genetic risk for intervention trial), the frequency of depressive or anxiety symptoms was significantly lower at follow-up visits [41]. One article reported improved diabetes-specific quality of life for children, and reduced frequency and difficulty in parenting stress for children diagnosed with stage 3 type 1 diabetes following screening participation compared with matched community controls (birth cohort). However, the mean parental SAI score was elevated (>40) at the time of stage 3 diagnosis, and there was no significant difference in parental diabetes-specific anxiety in the screened cohort compared with the community controls in the first year thereafter [43].

Conversely, five articles suggested that anxiety can persist or recur [13, 24, 25, 31, 33]. In ASK, 6 months after results notification, despite a significant decrease in the mean anxiety level, anxiety remained above the clinical threshold in 69% of parents [13]. In TEDDY (birth cohort), 47% of mothers had clinically relevant anxiety (SAI >40) at study commencement, with significantly more mothers than fathers displaying high anxiety (47% vs 34%, *p*<0.0001). During follow-up, 57% of mothers and 44% of fathers displayed high anxiety (SAI >40) 1 year after multiple persistent positive notification, compared with 43% of mothers and 34% of fathers after 3 years. Maternal anxiety was strongly associated with persistent single or multiple Aab positivity whereas paternal anxiety was strongly associated with multiple Aab positivity [31]. In DIPIS, having a child positive for one or more Aabs was associated with higher anxiety (SAI >40) compared with having an Aab-negative child [33]. Up to 12 years after notification, qualitative interviews confirmed that parents experienced occasional lingering concerns, particularly around monitoring visits or symptom emergence [24, 25].

Characteristics associated with anxiety were assessed by evaluating 13 quantitative articles that performed regression analyses. Four subgroups with heightened anxiety were identified: (1) FHx [29, 31, 33, 36, 37, 42], (2) pre-morbid anxiety or depression [25, 29, 31, 33, 36, 40, 44], (3) multiple Aabs compared with single Aab or extremely high genetic risk [14, 29, 31–34] and (4) demographic subgroups including ethnic minorities, lower parental education level and single mothers [13, 29, 31, 34, 37, 40, 42].

#### Theme 2: cognitive implications of screening

The following sub-themes were identified: initial accurate risk perception; risk interpretation varied; accurate risk perception declined; and characteristics associated with accurate risk perception.

In the assessment of risk perception, the four quantitative articles from the ASK and PANDA studies and one qualitative article (KEA) demonstrated initial accurate risk perception for high-risk children, with 67–79% of parents estimating risk at a level aligned with clinical probability within 1 month of notification [13, 24, 35, 42, 45]. For parents of children with low genetic risk, 92% accurately thought their child’s risk was less than/equivalent to the background population at 16 weeks and up to 1 year after notification [14]. For children aged 10 years, 40% were unsure of their risk status, 31% underestimated their risk and 29% accurately understood they were at increased risk; for children aged 15 years, 14% were unsure, 45% underestimated their risk and 42% had accurate increased risk perception [46].

Five quantitative articles, including PANDA and TEDDY, showed that risk accuracy waned with time, leading to risk underestimation [33, 35, 42, 45, 47]. Within 6 months of high-risk notification, 50–87% of parents correctly estimated risk, while 8–24% underestimated their child’s risk [14, 35, 45]. Two qualitative articles (KEA) found parents either forgot results or were reassured by their child’s good health [24, 25].

The present meta-synthesis of two qualitative and six quantitative articles showed that the perceived likelihood of developing type 1 diabetes differed between parents and intra-individually over 12 years follow-up [14, 24, 25, 27, 28, 32, 34, 35] and that risk comprehension was associated with uncertainty [14, 24, 27, 28, 32, 45]. Qualitative interviews suggested that understanding risk was a dynamic process which involved the parent locating their child's diabetes risk status on a map of relative risks, potential illnesses or situations they considered as more serious or probable, such as injuries (falls), childhood cancer, drowning, poor diet and being upset at day care [24, 25].

Characteristics associated with accurate risk perception were assessed using 14 articles [13, 29, 31, 33, 34, 36, 37, 42, 44–49]. Significant factors associated with accurate risk perception were identified, including FHx status (FHx mothers/fathers compared with general population) [13, 47] and high-risk classification (multiple vs single Aab positivity) [13]. Mothers with higher anxiety levels had more accurate risk perception and overestimation of risk associated with increased anxiety levels [13, 29, 31, 33, 34, 37, 42, 45]. Conversely, minority ethnicity, lower educational level and deprivation predicted lower risk accuracy [13, 47]. Children were significantly more likely to have accurate high-risk perception if the following applied: child was of older age; child had positivity for one or more Aabs; mother had a college education; mother had more accurate risk perception; study was from a US centre compared with Europe; child was female; and child had a first-degree relative with type 1 diabetes [46].

#### Theme 3: behavioural implications of screening

The following sub-themes were identified: behavioural strategies; monitoring behaviours; lifestyle changes; restrictive behaviours; and characteristics associated with behavioural implications.

Behavioural strategies were examined. Ten articles explored behavioural implications of screening [24–26, 28, 32, 34, 44, 48–50], including KEA, BABYDIAB (birth cohort) and TEDDY. Following high genetic risk notification, most parents (67–73%) endorsed at least one behavioural strategy [24, 49, 50], 30% reported 2–3, 24% reported 4–6 and 8% reported >6 behavioural strategies [50]. In TEDDY, 6–15 months following high-risk notification, 30–43% of mothers reported one or more behaviour(s) to prevent type 1 diabetes [48].

Evaluation of monitoring behaviours revealed that the most frequently reported actions following high genetic risk notification or participation in Aab surveillance were those recommended in accordance with current international guidelines [11], including symptom monitoring and home glucose testing [24, 25, 44, 49–51]. Parents with FHx performed monitoring behaviours significantly (19 times) [50] more often than general population families [24, 25, 44, 50, 51] and mothers more than fathers [44]. Qualitative articles showed that parents frequently considered type 1 diabetes when the child was unwell but infrequently employed health-seeking behaviour for reassurance [24].

In TEDDY, FHx parents performed monitoring behaviours and glucose monitoring more frequently than general population parents. Following first Aab detection, monitoring and glucose monitoring behaviours increased for both FHx parents and general population parents but the increase was higher for FHx parents. Similarly, monitoring behaviours were significantly more frequent for Aab-positive children than for Aab-negative children [44].

Lifestyle changes were assessed using six articles that explored lifestyle changes after risk notification [24, 25, 28, 44, 48, 50], including KEA and TEDDY. Here, lifestyle changes were endorsed or undertaken by 14–34% of parents, including diet modification (reducing sugar), prolonged breast feeding and increased physical activity [48, 50]. Qualitative studies indicated that high-risk notification was an incentive to lead as healthy a lifestyle as possible for the whole family [24]. Despite this, some families admitted not making lifestyle changes (or at least not due to the child’s high-risk status) and those that implemented changes did so regardless of their child’s risk [24, 25, 28, 50].

Seven articles examined restrictive behaviours [24–26, 34, 41, 49, 50], including KEA and TEDDY. Up to 3 years after risk notification, a minority of parents (1–9%) performed restrictive behaviours [50], such as foregoing more children [26], administration of medications or vitamin supplementation, illness prevention (e.g. limiting exposure to other children), stress reduction or change to the child’s intended profession [24, 25, 34, 49, 50].

Characteristics associated with behavioural implications were examined. Significant factors associated with behavioural modifications at 6 months included older maternal age, higher maternal education, only one child, FHx, higher anxiety or postpartum depression, accurate risk perception and belief that risk was modifiable. The strongest factors associated with monitoring behaviours included FHx parents and accurate risk perception compared with parents who underestimated their child’s risk [44].

#### Theme 4: acceptability of screening

The meta-synthesis identified the following sub-themes for acceptability of screening: motivation for early detection; reservations about early detection; and participation experience and preferences. In total, 39 articles explored acceptability of participation in a type 1 diabetes screening/testing programme, including nine qualitative, 29 quantitative and one mixed methods [24, 27, 28, 30, 34, 38, 51–83]. ESM Table 6 displays the positive and negative indicators of acceptability.

Motivation for early detection was assessed from 21 articles [24, 33, 34, 49, 50, 53, 55, 56, 58–60, 62, 64, 65, 69, 75, 77–81], including KEA, ABIS, TEDDY and T1Early (gen pop, Aab). In common across all study types, parents saw benefit for them and their child, including having time to prepare, information gathering and forward planning for a smoother transition to stage 3 disease [24, 75, 77]. Parents valued monitoring to ‘watch over’ the child for development of type 1 diabetes [33–56, 60, 65] and the possibility of prevention strategies [49, 50, 69]. Parents particularly valued ascertaining their child’s risk information when type 1 diabetes or other chronic conditions (allergy/coeliac disease) ran in the family [59, 64, 77, 78, 81].

Parents of Aab-positive children frequently adhered to follow-up programmes (e.g. 68–76% adherence to Aab surveillance [63]). The mean adherence to oral glucose tolerance testing was 62% in TEDDY, ranging from 48% to 78% across TEDDY international centres and Trialnet (FHx, Aab) [55, 76].

Five hypothetical articles assessed screening acceptability [56, 72–74, 82]. Parents considered screening more acceptable when combined with prevention trials or treatment [56, 73]. A DCE survey revealed that screening experience (cost, invasiveness) was less important to parents than availability of an effective treatment [56]. This shows hypothetical–empirical confirmation, as demonstrated bycombined willingness to prevent type 1 diabetes (hypothetical) and up to 43% of mothers implementing a preventative behavioural strategy (empirical evidence) [48].

Reservations about early detection were examined using 13 articles highlighting disadvantages or reasons for non-participation in type 1 diabetes testing or birth cohort studies [24, 38, 53, 58–61, 63, 64, 66, 69, 75, 77], including KEA, ABIS, T1Early and TEDDY. The main concern was discomfort from blood draws, more so in fathers [64], emphasised by 19% of general population parents and 14% of FHx parents in TEDDY [64] and up to 36% of parents in ABIS [38, 58, 60, 69, 77]. Other issues included lack of time, travel burden and language barriers [38, 53, 58, 64]. Parents worried about the implications of testing, such as not wanting to deal with a ‘positive’ result [24, 60, 64, 77]. Perceived irrelevance (no FHx) [58, 64], lack of information and fathers’ lack of agreement also contributed [38, 58, 69, 77]. This was confirmed in qualitative interviews (ABIS) with six parents who declined participation [69, 77].

The majority (72%) of families in the TEDDY study remained in follow-up. Here, reasons for withdrawal from long-term monitoring were provided in seven articles [30, 53, 60, 61, 63, 64, 66], and included concerns about blood draw and lack of time and 27% were passive withdrawals (non-responders) [53, 60, 63, 66].

Three hypothetical articles reported perceived social implications, including medical insurance concerns, raised by 91% in a focus group study [73, 74] and 61% feared that test results could have implications on parent–child bonds or affect other family members [72, 73].

Participation experience and preferences were assessed from 22 articles [24, 26–28, 33, 34, 37, 51, 52, 54, 57, 58, 62, 65–71, 75, 77], across all study types, including KEA and ABIS, BABYDIAB, Fr1da, Type1Screen (FHx, Aab), UNISCREEN (general population, Aab), T1Early, Trialnet and Point/GPPAD.

In UNISCREEN, over 80% of children thought the screening would be useful to prevent future health problems and could improve the child’s health and quality of life, and would support type 1 diabetes population screening [52]. Regarding study satisfaction, in TEDDY, parents with an accurate risk perception and who believed something could be done to prevent type 1 diabetes had higher study satisfaction scores, whereas more-educated parents and those with higher depression scores displayed lower satisfaction. Staff consistency was associated with satisfaction in Europe but not in the USA [71].

A hypothetical qualitative study showed that parents preferred the ease and convenience of home sampling for the screening test [57] and considered peer support a helpful resource to support the process of coming to terms with an early-stage type 1 diabetes diagnosis, particularly to follow on from the screening programme and continue through to stage 3 disease [68].

Blood sampling was considered burdensome [24, 75, 77] (e.g. by 15% of parents and 48% of children in BABYDIAB [26]). A US study (Aab) showed that children initially regretted participation, although this fell to 10% at follow-up [27, 28]. In Trialnet, parents and children preferred less-invasive testing such as capillary sampling at home [67]. Overall, parents’ satisfaction was high [24, 70], exceeding 80% for detection studies [24, 33, 34, 37, 51, 58, 62, 65, 84]. Across population genetic and Aab testing programmes (KEA, ABIS, T1Early), parents perceived benefits to outweigh harms [69, 75] and 12 years after screening, parents perceived no adverse effects (KEA) [24].

#### Theme 5: research ethics of screening

Overall, 19 articles [24, 26, 34, 37, 38, 51, 52, 59, 69, 75, 77–81, 84–87] explored research ethics considerations, including five qualitative and 14 quantitative articles, from the ABIS, Fr1da and UNISCREEN studies. The meta-synthesis identified three sub-themes: decision making; results disclosure; and data integrity.

Decision making and informed consent was discussed in ten articles [37, 51, 52, 77, 79–81, 84–86]; all except two related to the ABIS study (Fr1da and UNISCREEN). Almost all parents (89–90%) and children (83%) felt there was sufficient information to make an informed [37, 51, 77, 79, 80, 84] and voluntary (95–100%) decision [77, 84]. In UNISCREEN, >80% of parents and children said they understood what to do following a positive screening test and understood the overall programme [52]. ABIS reported that parents raised no concerns with surrogate or best interests’ decision making [77]. Yet, 58% of parents thought children should have a right to decide whether to participate (when aged >8 years) [60] and 70% upheld the right to dissent to blood taking [85]. Nearly all children (85–92%) wanted to be involved in decision making and most (53%) preferred joint decision making with their parents [80, 81].

Ten articles reported results disclosure preferences [24, 26, 34, 38, 59, 75, 77, 78, 86, 87], mostly from the KEA and ABIS studies but also from BABYDIAB and T1Early. Nearly all parents (90-100%) wished to be informed of the child’s risk [26, 34, 59, 77] and 74–87% wished to know the results despite lack of a prevention agent [59, 77, 87].

The genetic risk population studies showed that parents expressed the right to know the results in exchange for research participation [77]. Here, most parents (66–98%) thought that the child should receive individualised risk information (when aged >8 years) [59, 85]. Both parents and children wanted results delivered in a person-centred manner, by experienced professionals, and welcomed pedagogically designed children’s resources [24, 79, 81].

Data integrity and handling was discussed in eight articles [59, 77–80, 84–86], all but one pertaining to the ABIS study. Parents emphasised confidentiality, with access limited to researchers pursuing the detection study’s intended aims. Parents also stressed the importance of data integrity, with safeguarding to avoid data violation and data use for the purposes for which the consent was achieved [59, 78, 84, 85].

## Discussion

This is the first systematic review to assess the psychosocial implications, acceptability and research ethics of early detection for type 1 diabetes. This synthesises 30 years of evidence using a mixed methods approach, thus allowing a large pool of heterogeneous studies to be assessed. Overall, there is high-quality evidence of psychosocial impact on parents/guardians following detection of early-stage type 1 diabetes. Although there is insufficient evidence to judge whether population screening is broadly acceptable or ethical, the early indicators for parents’/guardians’ screening acceptability, in terms of satisfaction level and perceived benefits vs harms, are positive.

There are five major findings. First, there is initial heightened anxiety after positive results notification and anxiety remains high for mothers with a multiple-Aab-positive child. Demographic and clinical factors associated with anxiety include FHx, maternal pre-morbid anxiety, later stages in the natural history and specific underserved groups. Second, parents generally understand the implications of positive results but risk accuracy could decrease with time. Third, screening results can stimulate behavioural changes, many of which align with monitoring guidelines, such as increased home glucose testing. Some families endorse lifestyle changes and restrictive behaviours are reported rarely. Fourth, motivations for screening include parents wanting to know if their child has early-stage type 1 diabetes or is genetically predisposed, and to benefit from time to prepare and follow-up, while the main reservation is blood taking. Overall, screening appears to be acceptable but perhaps would be more acceptable if disease-modifying therapies were available. Fifth, detection studies meet parents’ expectations for research, with sufficient information for valid informed consent and maintenance of confidentiality. While we offer summary estimates regarding overall acceptability, there is insufficient evidence to make direct comparisons between the acceptability of genetic and Aab screening or general population and FHx screening [4, 29, 64].

Critically, the 30 year span of evidence has seen a rapid evolution in our understanding of early-stage type 1 diabetes [88]. Results communication to parents has varied widely between the genetic and Aab screening and early type 1 diabetes detection studies, making comparisons over time between psychosocial and acceptability outcomes extremely challenging. For example, the ABIS study (1997–1999) only released Aab results on request and was associated with limited parental concerns regarding screening [38, 69, 78]. Conversely, recent large general population screening programmes, principally Fr1da (2015–present) and ASK (2017–present), inform parents of near lifetime certainty of type 1 diabetes, contributing to the observed parental anxiety [4, 55].

Our review supports previous narrative reviews in this area regarding emotional impacts, difficulties comprehending risk, and behaviour changes aiming to smoothen the transition to stage 3 [2, 10, 89]. Nevertheless, inclusion of qualitative articles has expanded our understanding, with additional findings summarised in ESM Table 7. For example, emotional implications may persist or recur relating to symptom emergence, and parents’ risk interpretation varies, displaying both over- and underestimation over time. While early detection of type 1 diabetes is associated with anxiety and stage 3 diagnosis is recognised as life-changing [90], structures are needed to address the negative psychosocial impact that arises from screening, through psychological input [4] and possibly peer support [68]. These structures should identify families with persistent anxiety who display maladaptive behaviours and negative coping strategies. Systems also need to support the clinical care of children identified with early-stage type 1 diabetes and educate their parents/guardians to ensure appropriate disease perception. It is imperative that parents/guardians understand the importance of monitoring, particularly in the advent of potential disease-modifying therapies, as a child becomes eligible for a treatment or trial when progressing from stage 1 to stage 2.

The major novel finding is that parents who opted into a type 1 diabetes screening/early detection research programme perceived the benefits of knowing their child’s risk to outweigh potential harms (ESM Table 7). The evidence for this includes high levels of satisfaction with screening/detection programmes, significant uptake for screening (recruiting to target), parents valuing knowledge of their child’s genetic risk of type 1 diabetes and/or detection of early-stage disease providing opportunities to prepare, monitor and access prevention strategies. Further, detection studies have not raised concerns in relation to research ethics principles. These are important insights and are similar to findings observed following early detection of other chronic paediatric conditions [91, 92] where parents favour access to information despite the psychosocial implications of living with risk [11, 93–97]. Burdens of screening include paediatric blood sampling and commitment to monitoring visits for ongoing testing. This likely contributes to the parental anxiety observed during follow-up and discontinuation from monitoring. However, these findings relate to birth cohort studies, when optimal follow-up had not been established. As early-stage type 1 diabetes is now a recognised disease state [88] with ICD-11 and SNOMED codes and consensus guidelines for follow-up [11], population screening programmes must re-evaluate monitoring adherence and reasons for withdrawal.

We present important insights that can be taken forward to increase screening acceptability. First, screening should where possible incorporate less-invasive testing, such as capillary sampling [56, 67, 74]. Second, parents would prefer to participate in a screening programme if there was a licensed disease-modifying treatment for children identified with early-stage type 1 diabetes [24, 56, 72]. This is supported by international quantitative and qualitative articles that demonstrate high acceptability and interest in disease-modifying therapies [98]. Teplizumab is currently licensed in the USA, UK and Europe, and other clinical trials, including lifestyle-based trials for prevention are emerging [7]. As these disease-modifying therapies become more widely available worldwide, it is prudent to re-evaluate the benefits, risks and overall acceptability of paediatric type 1 diabetes screening.

**Fig. 3:**
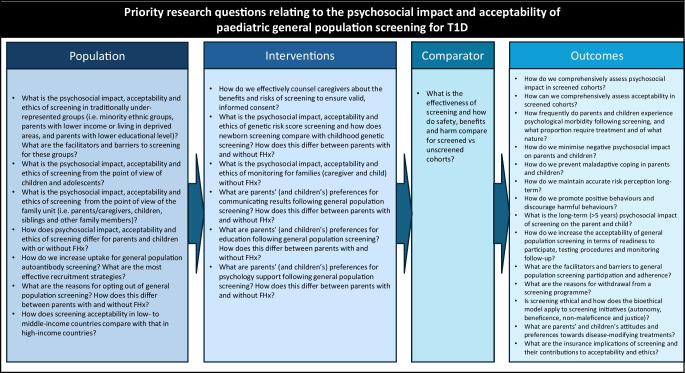
Priority research questions for paediatric, general population type 1 diabetes screening. T1D, type 1 diabetes

This review’s strengths are its comprehensive search strategy and mixed methods evidence synthesis, summarising the available international evidence to date. However, there are several limitations. First, there was substantial heterogeneity in study designs (e.g. Aab vs genetic screening) and the included populations differed (FHx vs general population), with under-representation of minority ethnic groups and parents with a lower educational level, and studies were primarily conducted in high-income countries. Second, consensus guidelines and analytical frameworks are lacking on how to assess psychosocial impact, acceptability and ethics in screened cohorts. While validated measures were used, attempts to separate anxiety associated with screening from concurrent life stresses is extremely difficult and potentially futile due to their bidirectional nature; it is not possible to determine the extent to which anxiety about a child developing type 1 diabetes creates the stressor as compared with exacerbation of existing life stressors. This, combined with lack of randomised studies comparing screened to unscreened cohorts, confers high-risk of bias and precludes causal interpretation. Third, most studies assessed impact up to 12 months following diagnosis and only TEDDY assessed anxiety up to 5 years after detection [33]. Therefore the longer-term impacts of screening on the parent or growing child are relatively unknown. Unfortunately, the prevalence and clinical severity of anxiety remains unclear and the consequences of heightened anxiety, including need for psychological input or medication, are not reported. Fourth, screening procedures differed and access to prevention opportunities varied, and this will have influenced experience and perceived acceptability. The acceptability data are from the subset of the population who opted into screening research and completed an acceptability assessment metric. Broader understanding of population-level acceptability, particularly for under-represented groups and those opting out of screening, is needed to address concerns and improve equitable access. Additionally, there is a clear need to harmonise screening efforts and assessment tools. Qualitative interview studies are lacking, particularly for general population Aab screening, but are essential to understand rationale, psychosocial impacts and breakdown of what is and is not acceptable. Finally, relatively few articles assessed children’s views (*n*=12), meaning that psychosocial impact and acceptability could not be ascertained for the primary participant. The aforementioned weaknesses are significant unmet needs, which future research programmes, such as EDENT1FI, will aim to address. Here, different psychosocial assessment tools will be compared, including the SAI, Hospital Anxiety and Depression Scale and the EuroQuol-5d, qualitative interviews will be performed across European countries and children’s views will be explored. Finally, Fig. 3 outlines the priority research questions pertaining to general population, paediatric type 1 diabetes screening, to help direct future research programmes.

This mixed methods systematic review is the largest and most comprehensive of its kind in this field. Despite clear psychosocial impact to parents/guardians of diagnosing early-stage type 1 diabetes, there are early indicators that support parental acceptability, caveated by significant evidence gaps, particularly regarding the impact on children. To advance the field, we outline important considerations for general population screening programmes and provide a structure with which future investigations in this area can proceed.

## Supplementary Information

Below is the link to the electronic supplementary material.

ESM (PDF 781 KB)

ESM Table 5 (XLSX 49 KB)

## Data Availability

The datasets generated during this study are available from the corresponding author upon reasonable request.

## Abbreviations

Aab: Autoantibody
CASP: Critical appraisal skills programme
DCE: Discrete choice experimental
DKA: Diabetic ketoacidosis
FHx: Family history of type 1 diabetes
MMAT: Mixed Methods Appraisal Tool
PHQ-9: Patient Health Questionnaire 9
PRISMA: Preferred Reporting Items for Systematic Reviews and Meta-Analyses
SAI: State anxiety inventory
TFA: Theoretical framework of acceptability
