# Supplementary material for: Psychosocial implications, acceptability and ethics of screening for paediatric type 1 diabetes: a systematic review and mixed methods evidence synthesis
Source: Diabetologia. 2026 May 1;69(7):1759–81. doi: 10.1007/s00125-026-06717-2 (PMC13236767; doi:10.1007/s00125-026-06717-2)
Supplement: Supplementary file 1 — ESM (PDF 781 KB) [file 125_2026_6717_MOESM1_ESM.pdf]

Electronic Supplementary Materials (ESM)

Table of Contents

**ESM Methods: Database searches**	**pages 2-9**
**ESM Table 1 – Inclusion and exclusion criteria**	**pages 10**
**ESM Table 2 – Study characteristics**	**pages 11-30**
**ESM Table 3 – Excluded articles and reasons for exclusion**	**pages 31-32**
**ESM Table 4 – Quality assessment**	**pages 33-36**
**ESM Table 5 – Full text data extractions**	**See Excel Spreadsheet**
**ESM Table 6 – Positive and negative indicators of acceptability**	**pages 37-48**
**ESM Table 7 – Summary of findings**	**pages 49-51**

ESM Methods: Database searches

1) **Medline**

Set	Search Statement
1.	exp Diabetes Mellitus, Type 1/
2.	screen.mp.
3.	screening.mp. or exp Mass Screening/
4.	detection.mp.
5.	surveillance.mp. or exp Public Health Surveillance/
6.	genetic.mp. or exp Genetics/
7.	exp Antibodies/ or antibody.mp.
8.	exp Qualitative Research/
9.	exp Interview/
10.	focus group.mp. or exp Focus Groups/
11.	questionnaire.mp. or exp "Surveys and Questionnaires"/
12.	survey.mp.
13.	view.mp.
14.	exp Attitude/
15.	experience.mp.
16.	decision.mp. or exp Decision Making/
17.	acceptability.mp. or exp "Patient Acceptance of Health Care"/
18.	exp Psychology/
19.	psycho.mp.

20.	exp Health Behavior/ or exp Health Knowledge, Attitudes, Practice/
21.	exp Knowledge/
22.	understanding.mp. or exp Comprehension/
23.	social.mp.
24.	exp Depression/
25.	exp Test Anxiety/ or exp Test Anxiety Scale/ or exp Anxiety/
26.	exp Health Behavior/ or health behaviour.mp.
27.	exp "Quality of Life"/
28.	2 or 3 or 4 or 5 or 6 or 7
29.	8 or 9 or 10 or 11 or 12 or 13 or 14 or 15 or 16 or 17 or 18 or 19 or 20 or 21 or 22 or 23 or 24 or 25 or 26 or 27
30.	1 and 28 and 29

02.07.2024 Results = 3335
25.11.2025 Results = 313

2) EMBASE

Set	Search Statement
1.	type 1 diabetes.mp.
2.	screen.mp.
3.	exp mass screening/ or exp screening/
4.	detection.mp.
5.	surveillance.mp.
6.	genetic.mp. or exp genetics/
7.	exp antibody/
8.	exp experience/
9.	exp decision making/ or decision.mp.
10.	view.mp.
11.	exp attitude/
12.	acceptability.mp. or exp program acceptability/
13.	exp qualitative research/
14.	exp interview/
15.	focus group.mp.
16.	questionnaire.mp. or exp questionnaire/
17.	survey.mp.
18.	exp psychology/
19.	psycho.mp.
20.	social.mp.
21.	health behaviour.mp. or exp health behavior/

22.	exp anxiety/
23.	exp test anxiety/
24.	exp depression/
25.	exp knowledge/
26.	understanding.mp. or exp comprehension/
27.	exp "quality of life"/
28.	2 or 3 or 4 or 5 or 6 or 7
29.	8 or 9 or 10 or 11 or 12 or 13 or 14 or 15 or 16 or 17 or 18 or 19 or 20 or 21 or 22 or 23 or 24 or 25 or 26 or 27
30.	1 and 28 and 29

02.07.2024 Results = 4800
25.11.2025 Results = 786

3) PsychINFO

Set	Search Statement
1.	exp Diabetes/ or type 1 diabetes.mp.
2.	exp Screening/ or screen.mp.
3.	exp Health Screening/ or mass screening.mp.
4.	detection.mp.
5.	surveillance.mp.
6.	exp Antibodies/ or antibody.mp.
7.	exp Genetics/ or genetic.mp.
8.	view.mp.
9.	attitude.mp. or exp Attitudes/
10.	experience.mp.
11.	decision.mp. or exp Decision Making/
12.	acceptability.mp.
13.	qualitative research.mp. or exp Qualitative Methods/
14.	exp Interviews/ or interview.mp.
15.	exp Focus Group/
16.	questionnaire.mp. or exp Questionnaires/
17.	survey.mp.
18.	exp Psychology/
19.	psycho.mp.
20.	social.mp.
21.	exp Health Behavior/ or health behaviour.mp.

22.	knowledge.mp.
23.	understanding.mp. or exp Comprehension/
24.	exp Anxiety/ or exp Test Anxiety/
25.	depression.mp.
26.	exp "Quality of Life"/
27.	2 or 3 or 4 or 5 or 6 or 7
28.	8 or 9 or 10 or 11 or 12 or 13 or 14 or 15 or 16 or 17 or 18 or 19 or 20 or 21 or 22 or 23 or 24 or 25 or 26
29.	1 and 27 and 28

02.07.2024 Results = 1146
25.11.2025 Results = 73

4) ASSIA

(type 1 diabetes) (Topic) and (screening OR screen OR detection OR surveillance OR genetic OR antibody) (All Fields) and (qualitative research OR interview OR focus group OR survey OR questionnaire OR view OR attitude OR experience OR decision making OR psychology OR psycho OR social OR health behaviour OR knowledge OR understanding OR comprehension OR anxiety OR depression OR acceptability OR quality of life) (All Fields)

Up till 02.07.2024 Results = 247
25.11.2025 Results = 22

5) Web of Science

TS=("Type 1 diabetes" OR "diabetes mellitus, type 1") AND
TS=(Screen OR Screening OR "Mass screening" OR Detection OR surveillance OR Genetic OR genetics OR Antibody OR antibodies) AND
TS=(Acceptability OR "Patient acceptance of healthcare" OR acceptability OR "Qualitative research" OR Interview OR "Focus groups" OR "focus group" OR "Surveys and questionnaires" OR survey OR questionnaire OR Experience OR "decision making" OR Views OR Attitude OR Psychology OR psycho OR "Health behaviour" OR "health knowledge" OR "attitudes/practice" OR "Test anxiety" OR "test anxiety scale" OR anxiety OR Depression OR Social OR Knowledge OR comprehension OR Understanding OR "Quality of life" OR Retention OR compliance OR adherence OR withdrawal OR Uptake OR Recruit OR Enrol OR Concern OR issue OR fear OR "Bioethical issues")

02.07.2024 Results = 3968
25.11.2025 Results = 727

6) SCOPUS

("Type 1 diabetes" OR "diabetes mellitus, type 1") AND
(KEY(Screen OR Screening OR "Mass screening" OR Detection OR surveillance OR Genetic OR genetics OR Antibody OR antibodies)) AND
(KEY(Acceptability OR "Patient acceptance of healthcare" OR acceptability OR "Qualitative research" OR Interview OR "Focus groups" OR "focus group" OR "Surveys and questionnaires" OR survey OR questionnaire OR Experience OR "decision making" OR Views OR Attitude OR Psychology OR psycho OR "Health behaviour" OR "health knowledge" OR "attitudes/practice" OR "Test anxiety" OR "test anxiety scale" OR anxiety OR Depression OR Social OR Knowledge OR comprehension OR Understanding OR "Quality of life" OR Retention OR compliance OR adherence OR withdrawal OR Uptake OR Recruit OR Enrol OR Concern OR issue OR fear OR "Bioethical issues"))

02.07.2024 Results = 1631
25.11.2025 Results = 120

7) CINAHL

(type 1 diabetes) (Topic) and (screening OR screen OR detection OR surveillance OR genetic OR antibody) (All Fields) and (qualitative research OR interview OR focus group OR survey OR questionnaire OR view OR attitude OR experience OR decision making OR psychology OR psycho OR social OR health behaviour OR knowledge OR understanding OR comprehension OR anxiety OR depression OR acceptability OR quality of life) (All Fields)

02.07.2024 Results = 211
25.11.2025 Results = 186

ESM Table 1 – Inclusion and exclusion criteria

	**Inclusion criteria**	**Exclusion criteria**
Population	If participated in a screening study:<br>1. Parents/ carers/ caregivers/ guardians of a child screened for type 1 diabetes, with or without a family history of type 1 diabetes<br>2. Child/children screened for type 1 diabetes, aged <18 years, with or without a family history of type 1 diabetes<br><br>Hypothetical (If not previously participated in a screening study):<br>1. Parents/ caregivers, with or without a family history of type 1 diabetes<br>2. Child/children aged <18 years, with or without a family history of type 1 diabetes	1. Adult screening (≥18 years)<br>2. Children with type 1 diabetes or their parents<br>3. Screening for type 2 diabetes
Intervention	Screening or early detection programme for type 1 diabetes:<br>1. Genetic testing<br>2. Antibody testing<br>3. Birth cohorts (combined genetic and antibody genetic)<br>4. Detection with prevention trial	
Comparator	1. Usual care or unscreened cohort<br>2. Comparison group not a requirement for inclusion	
Outcome	Participated in screening<br>1. Acceptability – reasons for and against participation, screening experience, screening preferences<br>2. Implications of screening:<br>a. Emotional – psycho*, anxiety, test anxiety, depression, fear, concern<br>b. Cognitive – knowledge, comprehension, understanding, decision making<br>c. Behavioural – behaviour or health behaviour<br>d. Social<br><br>Hypothetical (not participated in screening):<br>1. Views, attitudes	No assessment of acceptability or implications of screening
Study designs	1. Qualitative (including but not limited to interviews, workshops or focus groups)<br>2. Quantitative (including but not limited to survey or questionnaire)	1. Validation studies for screening tests or outcomes measures<br>2. Psychological, behavioural or social risk factors for antibody seroconversion or type 1 diabetes diagnosis<br>3. Interventions to improve enrolment, retention or adherence to study follow-up

ESM Table 1 legend

Inclusion and exclusion criteria according to the Population, Intervention, Comparator and Outcomes (PICO) framework. Key: *=explode.

ESM Table 2 – Study characteristics

Author (reference)	County	Study design	Instrument	Aims	Population	Sample size (response rate, %, if reported)	Family history of T1D (%)	Intervention	Comparator (Control)	Timepoints assessed in relation to risk notification	Outcomes				
											Emotional	Cognitive	Behavioural	Other	Acceptability and ethics
Aas *et al.* 2010 (36)	Norway	Uncontrolled time series Pre and post questionnaire	Questionnaire	*"To assess whether mothers of children who test positively suffer from poorer mental health and well-being after receiving genetic risk information about their children."*	Mothers, General population	n=7390 provided first and second questionnaires (High (n=166) vs low risk (n=7224)) (response rate 80%)	0.5-0.6% had maternal T1D	MOBA/Midia - genetic newborn screening	No	Pre- 30th week pregnancy, Post - within 4m (6 m post-partum) (2)	Symptom Checklist (anxiety and depression) SCL-8, Rosenberg Self-Esteem Scale RSES, Satisfaction With Life SWL-5, Worry.				
Angiulli *et al.* 2025 (52)	Italy	Cross-sectional	Questionnaire	*"The aim of this study was to assess feasibility and acceptability of UNISCREEN, a population screening program across all ages."*	Children aged <18 years	n=242 children aged 0-15 years	NR for children only	UNIScreen – general population autoantibody screening	No	Pre and immediately post screening test					Acceptability questionnaire – attitudes and understanding
Baughcum *et al.* 2005 (50)	US	Uncontrolled time series - Structured telephone interview	Questionnaire (structured telephone interview)	*"To assess maternal diabetes prevention efforts aimed at children identified as at risk through newborn genetic screening."*	Mothers, General population	n=192 (extremely high risk n=13, high risk n=71, moderate risk n=108) (response rate 94%)	62% had a first or second degree relative with T1D, 26% no family history	PANDA - newborn genetic screening	No	Post - within 1m, 6m and 12m (3)	State anxiety inventory-10 (STAI)	Diabetes risk perception, perceived control, information seeking	Ways of coping checklist-20		
Baxter *et al.* 2012 (53)	US	Uncontrolled cohort	Questionnaire, Clinical research file	*"To investigate ethnic minority differences in patterns of enrolment and retention."*	Mothers and Fathers, Genetically predisposed child	n=3,242 enrolled, n=3,647 refused, and n=4,063 were excluded	Enrolled 45% FDR and 47% no FDR, Excluded 20% FDR and 38% no FDR	TEDDY - Genetic and Antibody surveillance	No	2005-2009					
Bendor-Samuel *et al.* 2023 (54)	UK	Uncontrolled cohort	Clinical research file	*"The aim of the INGR1D study (INvestigating Genetic Risk for type 1 Diabetes) was to implement a novel large-scale genetic research screening tool to identify a cohort of infants at an increased risk of early-onset T1D large enough to serve recruitment into the POInT trial."*	Mothers, General population	n=15,660 enrolled, 107 withdrawals	4% had FDR with T1D	INGR1D + POINT - GPPAD	No	04/2018-11/2020					Reasons for withdrawals, reasons for accepting or declining participation

Carmichael *et al.* 2003 (35)	US	Uncontrolled time series - Structured telephone interview	Questionnaire (structured telephone interview)	*"To assess mother's understanding of the infant's risk for developing type 1 diabetes."*	Mothers, General Population	n=435 first questionnaire, n=344 follow-up questionnaire (Extremely high (n=23, 5.3%), high risk (n=151, 34.7%), moderate risk (n=261, 60%)) (response rate 90%)	11% FDR, 61% SDR, 2% GDM, 25% no family history	PANDA - newborn genetic screening	No	Post - within 1m and within 6m (2)	STAI	Understanding and beliefs about risk			
Driscoll *et al.* 2021 (55)	US and Europe (Finland, Sweden, Germany)	Uncontrolled cohort	Questionnaire and Clinical research file	*"To examine adherence to the oral glucose tolerance test (OGTT) in multiple islet autoantibody children in stage 1 of developing type 1 diabetes (T1D)."*	Mothers, Genetically predisposed child	n=437 multiple antibody positive, ≥3 years of age	22% FDR	TEDDY - Genetic and Antibody surveillance	No	Child attended for >1 TEDDY study visit	STAI-20, wellbeing questionnaire	T1D risk perception and beliefs	Maternal actions		
Dunne *et al.* 2021 (56)	US	Cross-sectional, questionnaire, Discrete choice experimental survey	Questionnaire (Discrete choice experimental survey, online)	*"To understand the relative importance of the attributes of screening tests for type 1 diabetes among parents and pediatricians in the U.S."*	Mothers and Fathers, Children, General Population	n=1002 completed the survey	16% FDR, 84% no FDR	Hypothetical / attitudinal							
Faustini *et al.* 2025 (57)	UK	Qualitative (semi-structured interviews)	Qualitative (thematic analysis)	*"report the first acceptability data from semi-structured interviews for DBS as a sampling technique for T1D screening."*	Mothers, Fathers and children aged 3-13 years	n=33 families (n=38 parents and 13 children)	Parents - 30% FDR, Children – 27% FDR	Hypothetical / attitudinal	No						Perceived acceptability for the screening test, perceived screening test location preferences
Galatzer *et al.* 2001 (99)	Israel	Uncontrolled time series - questionnaire	Questionnaire	*"To evaluate the psychological impact of autoantibody screening and its results on at-risk individuals and family members."*	Mothers and Fathers, FDR screening	n=10 antibody positive children, n=15 parents (n=9 families)	100% FDR	Antibody screening	T1D siblings n=8	Post - immediately post-notification and within 3m (2)	Impact of Event Scale (Intrusion and Avoidance)				

Gesualdo *et al.* 2016 (58)	US	Cross-sectional, questionnaire	Questionnaire	*"To assess the feasibility of screening for IAs and tTGA in 2 to 6 yr-old children who were patients of a general paediatric practice."*	Mothers and Fathers, General Population	n=200 (26% completed same day screening and questionnaire)		Antibody screening (paediatric centre)	No	Post - immediate					Parental satisfaction, child's reaction to blood draw
Helgesson *et al.* 2008 (59)	Sweden	Cross-sectional questionnaire	Questionnaire	*"Families views on the handling of personal information, their expressed concerns, and how they view the need for renewed consents and how they want potential high-risk information about their participating child to be handled."*	Mothers and Fathers, General Population	n=7443 parents (NR response rate, 44% of those originally consented to ABIS)	NR	ABIS - newborn screening (not routinely informed of result)	No	Post- 5-6 years					(1) ‘‘How do you feel about the written information that you have supplied in questionnaires and ‘diary’?’’, (2) ‘‘How do you feel about the biological samples (blood, hair, breast milk, urine, etc.) that have been collected from you and your child?’’, and (3) ‘‘How do you feel regarding participation in the ABIS study?’’
Helgesson *et al.* 2009 (60)	Sweden	Cross-sectional questionnaire	Questionnaire	*"To investigate the importance of trust in researchers and other reasons that participating parents, former participants, and non-participants had for participating, or not participating, in a longitudinal cohort study on prediction and development of diabetes in children."*	Mothers and Fathers, General Population	n=1302 parents with children born between 1997-1999 (52% response rate)	NR FDR status	ABIS - newborn screening (not routinely informed of result)	No	Post - 7-9 years (2006)					Reasons for participating, reasons for opting out or dropping out, information and participation

Hendrieckx *et al.* 2002 (49)	Belgium	Cross-sectional, questionnaire	Questionnaire (self-administered)	*"To examine prospectively if participants in the screening programme for type 1 diabetes anticipated making changes in their daily life after being identified as having a high risk and whether they expected that these changes would reduce their risk."*	Mothers and Fathers, FDR screening	n=435 relatives of people with T1D (88% response rate)	Of children screened, 66% had a parent with T1D, 33% had a sibling with T1D	Antibody screening programme	No	Prior to risk notification	Wellbeing, concerns about developing T1D	Anticipated changes, perceived control over diabetes risk, perceived risk, perceived causes of diabetes, locus of control			Views on screening
Hommel *et al.* 2018 (100)	Germany	Uncontrolled, time series, questionnaire	Questionnaire (in-person)	*"We provide feasibility of the approach with preliminary results from screening in the first eight months of the study."*	Mothers and Fathers, General Population	n=56 at 4m and n=17 at 6m (76% response rate)	Of those screened, 1.3% FDR. Of those screened positive, 18% FDR.	Freder1k - newborn genetic screening, and Fr1da follow-up	No	Post - 4m and 6m	Psychological burden				
Hood *et al.* 2005 (40)	US	Uncontrolled time series - Structured telepone interview	Questionnaire (structured telephone interview)	*"The aim of this study was to examine the level of maternal depressive symptoms in response to the news that a child is genetically at risk for type 1 diabetes. Additional potential predictors of maternal response such as educational level, ethnic minority status, and coping style were examined."*	Mothers, General population	n=192 first questionnaire, n=144 second questionnaire (Extremely high (n=1, 0.5%), high (n=55, 29%) moderate risk (n=136, 71%)) (91% response rate)	7% FDR, 58% SDR, 35% no family history	PANDA - newborn genetic screening	Unmatched controls: CES-D normative sample 2514 individuals; CES-D data for 7600 mothers assessed 7m after childbirth	Post - 1m and within 6m (2)	Center for Epidemiologic Studies Depression Scale (CES-D-20), Edinburgh Postnatal Depression Score (EPDS-10)		Ways of coping checklist		

Hood *et al.* 2006 (45)	US	Uncontrolled time series - Structured telephone interview	Questionnaire (structured telephone interview)	*"We aimed to assess factors promoting maternal understanding of infant diabetes risk in a sample of mothers whose newborns had been identified as at increased risk for T1D."*	Mothers, General population	n=195 first questionnaire, n=144 second questionnaire (Extremely high (n=7, 4%), high (n=50, 26%), moderate risk (n=138, 71%) vs normative samples) (92% response rate)	9% FDR, 62% SDR, 29% no family history	PANDA - newborn genetic screening	Unmatched controls: Normative sample working adults - STAI and CES-D	Post - 1m and within 6m (2)	STAI-6, CES-D-20	Understanding and beliefs about risk			
Houben *et al.* 2022 (41)	Europe (Germany, Sweden, Belgium, Poland, UK	Uncontrolled cohort: Pre and post participation in POINT trial	Questionnaire	*"The aim is to explore potential differences in levels of depression and anxiety between mothers and fathers, between parents with or without a first-degree relative suffering from T1D and between the participating countries as well as exploring the emotional impact on parents over time."*	Mothers and Fathers, General Population	n=5730 questionnaires (n=2595 parents)	47.4% had T1D FDR	Freder1k and POINT - GPPAD	No	11/2017-05/2022	Wellbeing questionnaire: PHQ-9 (depression and anxiety total score), impact on daily life, expectation of increased risk and strain on mental health.				Feelings about participation
Hummel *et al.* 2004 (26)	Germany	Cross-sectional: Pre and post questionnaire	Questionnaire (mailed)	*"To determine anxiety in parents of children undergoing testing for islet autoantibodies."*	Mothers and Fathers, FDR screening	n=463 completed first questionnaire and n=317 completed second questionnaire (Of 260 invited families, n=249 Mothers and n=214 Fathers. Children were known antibody positive or negative before and after BABYDIAB visit	Mothers: 77% had T1D, 23% without T1D, Fathers: 25% with T1D and 75% without T1D	BABYDIAB - antibody surveillance	No	5 year visit: Pre and post blood withdrawal	STAI-20	Information seeking and worries linked to the children's diabetes risk	Reproductive intentions		Distress and burden for the parents and offspring related to the blood withdrawal;

Johnson *et al.* 1990 (27)	US	Uncontrolled time series - Structured telephone interview	Questionnaire (structured telephone interview)	*"To study participation, anxiety, and coping responses in ICA+ subjects and their family members."*	ICA+ children and at least one parent (excluded ICA+ adult dataset)	n=18 ICA+ children (8-17 years)	NR for children	Antibody screening	No	Post - immediate and within 2-4m (2)	STAI	Likelihood of developing T1D	Ways of coping checklist (coping response)		Feelings about participation
Johnson *et al.* 1995 (28)	US	Uncontrolled time series - Structured telephone interview	Questionnaire (structured telephone interview)	*"To describe the psychological impact of positive islet cell antibody (ICA) screening results in children and adults, as well as their parents and spouses."*	ICA+ children and parents	n=34 ICA+ children and n=33 parents (n=32 Mothers and n=1 Father)	NR	Antibody screening	No	Post - immediate and within 4m (2)	STAI and STAI for children		Behaviour changes made		Feelings about participation
Johnson *et al.* 2000 (101)	US	Uncontrolled time series - Structured telephone interview	Questionnaire (structured telephone interview)	*"How ICA+ve children and adults as well as family members coped with the news that they or a loved one was at risk for type 1 diabetes."*	ICA+ children (Excluded Mothers data)	n=37 ICA+ children and n=39 Mothers	NR	Antibody screening	No	Post - within 4m and within 10m (2)	STAI and STAI for children (STAIC)	Perception of controllability of ICA status	Ways of coping checklist and change in coping style overtime		
Johnson *et al.* 2004 (29)	US	Uncontrolled time series - Structured telepone interview	Questionnaire (structured telephone interview)	*"A longitudinal investigation of the impact of newborn genetic risk screening for type 1 diabetes on maternal anxiety levels during the year following risk notification."*	Mothers, General population	n=435 first questionnaire, n=344 follow-up questionnaire 1, n=269 follow-up questionnaire 2 (Extremely high (n=23, 5.3%), high risk (n=151, 34.7%), moderate risk (n=261, 60%)) (93% response rate)	14.3% FDR, 69% SDR, 26% no family history	PANDA - newborn genetic screening	Unmatched controls - 1) Parents of ICA+ children, 2) Pregnant women undergoing amniocentesis, 3) Pregnant women, 4) Working women	Post- within 1m, 4m, and 12m (3)	STAI-10	Risk and likelihood of T1D			

Johnson *et al.* 2011 (30)	Europe and US	Uncontrolled cohort	Questionnaire and Clinical research file	*"To identify predictors of early withdrawal from TEDDY among families with no immediate family history of T1DM."*	Mothers and Fathers, Genetically predisposed child	n=2994 families active ≥1 years vs n=763 who withdrew in the first year, general population only (FDR were excluded from analysis)	100% no FDR T1D	TEDDY - Antibody surveillance	No	Post -1 year follow-up					
Johnson *et al.* 2016 (61)	Europe and US	Uncontrolled cohort	Questionnaire and Clinical research file	*"To identify predictors of later study withdrawal among participants active in The Environmental Determinants of Diabetes in the Young (TEDDY) for 1 year."*	Mothers and Fathers, Genetically predisposed child	n=3042 active in first 3 years vs n=432 who withdrew in years 2 or 3, general population only (FDR excluded from analysis)	100% no FDR T1D	TEDDY - Antibody surveillance	No	Post - 2-3 years follow-up					
Johnson *et al.* 2017 (31)	Europe and US	Uncontrolled cohort	Questionnaire	*"To assess parent anxiety in response to genetic and islet autoantibody (IA) testing in children at increased genetic risk for type 1 diabetes."*	Mothers and Fathers, Genetically predisposed child	n=6799 (1-6 years antibody follow-up testing: parents of n=5985 Ab negative and n=814 Ab positive children followed for <1 year and n=718 children followed for 1-4 years	91% no family history of diabetes	TEDDY - Antibody surveillance	No	Post - within 3m, 6m and 15m and annually thereafter up to 6 years	SAI-6	Risk perception accuracy			
Kelly *et al.* 2024 (62)	US	Cross-sectional, questionnaire	Questionnaire	*"The purpose of this descriptive study was to fill this gap by beginning to understand knowledge, attitudes, and experiences with T1D autoantibody screening in a sample of individuals with a personal or close family history of T1D."*	Mothers and Fathers, Family history of T1D	n=216 (Parent/Guardian with a child with T1D and another child without T1D aged <18 years. Of these, n=100 had participated in antibody screening and n=116 had not (hypothetical). Other groups were excluded from analysis because these were mixed, including parents and adults with T1D or family history of T1D	100% FHx T1D	Any Antibody screening study (n=100) and Hypothetical/attitudinal (n=116)	No	NR					Acceptability and attitudes to T1D screening

Kerruish *et al.* 2007 (14)	New Zealand	Uncontrolled time series, questionnaire	Questionnaire	*"to evaluate maternal psychological reaction to receipt of genetic information concerning the newborn infant's risk of type 1 diabetes."*	Mothers and Fathers, General Population	n=38 Mothers with a high risk infant, n=76 Mothers with an age-matched low risk infant (93% response rate)	40% of high risk infants and 43% of low risk infants had a family history of T1D	KEA - cord blood genetic risk and antibody surveillance	n=76 Mothers with an age-matched infant not participated in screening	Post - within 3m, 5m and 13m	STAI-20, EPDS	Perception of infant's risk of developing T1D		Vulnerabl e Baby Scale (VBS)	Subjective rating of level of concern
Kerruish *et al.* 2011 (25)	New Zealand	Qualitative (semi-structured interviews)	Qualitative (semi-structured interviews: Interpretative phenomenologic al analysis)	*"To assess the psychosocial impact of screening newborns for genetic risk of type 1 diabetes."*	Mothers and Fathers, General Population	n=11 parents with a child at increased risk (9 interviews with the Mother and 1 interview with both parents) (67% response rate)	NR	KEA - cord blood genetic risk and antibody surveillance	No	Post - 2-3 years	Emotional reaction	Cognitive reaction	Lifestyle and behavioural changes		
Kerruish *et al.* 2016 (24)	New Zealand	Qualitative (semi-structured interviews)	Qualitative (Semi-structured interviews, thematic analysis)	*"To provide exploratory data concerning later effects through qualitative interviews with parents 12 years after newborn testing for T1D."*	Mothers, General Population	n=15 Mothers with a child at increased risk	20% had a relative with T1D (SDR or more distant)	KEA - cord blood genetic risk and antibody surveillance for ≈3 years	No	Post - 12 years	Emotional reaction	Cognitive reaction	Behavioural implications and monitoring		Disclosure to child, impact on child, assessment of benefits and harms, views toward genomics research
Kerruish *et al.* 2017 (32)	New Zealand	Cross-sectional, questionnaire	Questionnaire	*"To explore some of the more subtle and longer-term effects that genetic testing may produce in children."*	Mothers and Fathers, Children, General Population	n=35 parents high genetic risk child, n=64 parents low genetic risk child (86-92% response rate)	15% high risk infants had a T1D FDR and 35% had a family history of diabetes, 11% low risk had a T1D FDR and 38% had a family history of diabetes	KEA - cord blood genetic risk and antibody surveillance for ≈3 years	No	Post - 12 years		Perception of child's risk of developing T1D	Child behaviour Checklist, Alabama Parenting questionnaire		Subjective rating of level of concern, Disclosure of genetic test result to child,

Kick *et al.* 2019 (51)	Germany	Cross-sectional, questionnaire	Questionnaire	*"We aim to evaluate the study concept, feasibility and medical evidence of the Fr1da study."*	Mothers and Fathers, General Population	n=170 parents (66% response rate)	NR	Fr1da - antibody screening	No	Post - 6m		Educational training			Satisfaction, organisation of Fr1da and information provided
Kupila *et al.* 2001 (63)	Finland	Cross-sectional, questionnaire	Questionnaire	*"Feasibility and acceptance of neonatal genetic screening for Type I insulin-dependent) diabetes mellitus susceptibility and adherence of the at- risk children to frequent autoantibody follow-up were studied."*	Parents, General Population	n=4651 enrolled follow-up (80% uptake)	NR	DIPP - newborn genetic screening and surveillance	No	Post - 48m					Reasons for withdrawals
Lernmark *et al.* 2004 (37)	Sweden	Uncontrolled cohort	Questionnaire	*"To evaluate the impact on mothers and fathers both in the general population and in families with diabetes."*	Mothers and Fathers, General Population	n=10538 parents (n=6676 Mothers and n=6099 Fathers)	2.2% of participants had diabetes in the family	DiPiS - newborn genetic risk screening (informed about risk after 3 years)	No	Post - within 1-2 years	Concerns about child's risk of diabetes	Knowledge about diabetes pre-screening, risk perception of diabetes			Satisfaction with the information
Lernmark *et al.* 2011 (64)	Europe and US	Uncontrolled cohort	Questionnaire and Clinical research file	*"To identify characteristics of infants and their families who were enrolled, refused to enroll, or were excluded."*	Mothers and Fathers, General Population	n=6734 children parents enrolled, n=3837 children parents excluded, n=5864 children parents refused to participate	n=15329 no family history T1D, n=1106 T1D FDR	TEDDY - Antibody surveillance	No	2004-2009					Reasons for enrolment, exclusion and non-participation, satisfaction
Lernmark *et al.* 2012 (65)	Europe and US	Uncontrolled cohort	Questionnaire (in-person)	*"To assess parents' opinions about their participation in the longitudinal, multicenter study."*	Mothers and Fathers, General Population	n=2000 parents (59% response rate)	NR	TEDDY - Antibody surveillance	No	2008-2009					Reasons for adherence and suggestions for improvement, reasons for considering non-adherence

Lernmark *et al.* 2016 (66)	Europe and US	Uncontrolled cohort	Clinical research file (Change in study participation form)	*"To characterise participant reasons for withdrawing from a diabetes focused longitudinal clinical observational trial (TEDDY) during the first three study years."*	Mothers and Fathers, General Population	n=2109 describing first time reasons for withdrawal	NR	TEDDY - Antibody surveillance	No	Post- within 3 years					Reasons for withdrawal (active vs passive withdrawals)
Litchfield *et al.* 2024 (68)	UK	Qualitative (semi-structured interviews)	Qualitative (semi-structured interviews, secondary analysis, framework analysis)	*"a secondary, post hoc content analysis of data from a series of semi-structured interviews with parents involved in the study that explores the preferences for and potential of peer support delivered as part of any future T1D screening programme."*	Parents – mothers and fathers	n=33	30% FDR, 58% no family history	ELSA 1 hypothetical/ attitudinal	No						Preferences for peer support
Liu *et al.* 2017 (67)	UK	Cross-sectional, questionnaire	Questionnaire	*"To evaluate the feasibility of using self-collected capillary blood samples for islet autoantibody testing to identify risk in relatives of people with Type 1 diabetes."*	Children, aged 8-18 years, FDR screening	n=81 aged ≤8years and n=97 aged 9-18 years	100% FDR	Trialnet - Antibody screening	No	NR					Preference for capillary sampling or outpatient venepuncture, ease of testing
Ludvigsson *et al.* 2001 (38)	Sweden	Uncontrolled time series - questionnaire	Questionnaire	*"To explore if, and by how much, mothers in the general population become worried when their babies participate in a screening program."*	Mothers and Fathers, General Population	n=17055 participated in the screening (79% uptake) n=4948 questionnaires at 1 year	NR	ABIS - newborn screening (not routinely informed of result)	No	Post - within 3 days and within 12m					Reasons for non-participation, how feel about participation

Ludvigsson *et al.* 2002 (69)	Sweden	Qualitative (semi-structured interviews)	Qualitative (semi-structured interviews, common ethical theory)	*"To evaluate mothers' attitudes to and ethical views on participation in a research screening for prediabetes in an unselected birth cohort."*	Mothers and Fathers, General Population	n=21 (n=15 participated in ABIS, n=6 did not participate in ABIS)	NR	ABIS - newborn screening (not routinely informed of result)	No	NR					Attitudes towards the ABIS study, reasons for participation and non-participation, data storage and information provided
Melin *et al.* 2020 (33)	Sweden	Uncontrolled cohort	Questionnaire	*"To assess parental anxiety levels after 5 years of participation in the Diabetes Prediction in Skåne study."*	Mothers and Fathers, General Population, genetically predisposed children	n=2088 parents (n=2059 mothers and n=1933 Fathers), of children identified at risk followed for 5 years (n=1986 Ab negative, n=79 1 Ab and n=23 multiple Ab)	9% T1D FDR and 88% no family history of diabetes	DiPiS - antibody surveillance	No	Post - 5 years	SAI, frequency of worry about T1D	Risk perception of T1D			
Melin *et al.* 2022 (71)	Europe and US	Uncontrolled cohort	Questionnaire	*" The aim of the current study was to identify factors associated with parent study satisfaction in TEDDY, with a particular focus on the role of staff consistency."*	Mothers and Fathers, General Population, genetically predisposed children	n=5579 mothers and 4942 fathers (child aged 15 months), and n=4010 mothers and n=3411 fathers (child aged 48 months/four years)	14% T1D FDR	TEDDY – Antibody surveillance	No	Pre and post study intervention to reduce dropouts	6-item STAI, depression sub-scale of wellbeing questionnair e	Risk perception of T1D and belief the child's T1D risk can be reduced	Lifestyle changes		Parental study satisfaction questionnaire
Melin *et al.* 2023 (70)	Europe and US	Uncontrolled cohort	Questionnaire and Clinical research file	*"To identify sociodemographic variables and maternal characteristics assessed in the first year of TEDDY that were associated with study visit compliance in the subsequent 3 years."*	Mothers and Fathers, Genetically predisposed child	n=4600, enrolled and active (at least 1 visit per year at child aged 48 months)	9% T1D FDR, 91% no T1D FDR	TEDDY - Antibody surveillance	No	Post - 48 month visit					Predictors of adherence/compliance

Nicholls *et al.* 2013 (72)	Canada	Qualitative (workshops)	Qualitative (workshop, thematic analysis)	*The specific objectives of the present research were to explore, in participants drawn from the target populations for existing CRC and NBS programs, general reactions to the idea of incorporating genomic risk profiling into routine screening activities, and (ii) the most important issues requiring consideration as these technologies are assessed and implemented in practice."*	Parents with a child aged <5 years	n=50 participated across 8 workshops	NR	Hypothetical / attitudinal							
Nicholls *et al.* 2016 (73)	Canada	Qualitative (workshops)	Qualitative (workshop)	*"The primary aim of this study was to examine the attitudes of specific population target groups to the potential integration of hypothetical genomic profiling, using their experiences with existing screening programs as a starting point for deliberation."*	Parents with a child aged <5 years	n=40 parents	NR	Hypothetical / attitudinal							
O'Donnell *et al.* 2023 (13)	US	Uncontrolled, time series, questionnaire	Questionnaire	*"To assess anxiety and risk perception among parents whose children screened positive for islet autoantibodies, indicating elevated risk for type 1 diabetes (T1D)."*	Mothers and Fathers, General Population	n=280 caregivers (n=250 Mothers, n=24 Father and n=6 Other) with a child identified with ≥1 antibody	9% children had a T1D FDR, 91% no FT1D FDR	ASK - antibody screening and surveillance	No	Post -first visit and within 6m	SAI-6	Parent's risk perception			

O'Donnell *et al.* 2025 (46)	Europe / US	Uncontrolled cohort	Questionnaire	" To assess children's understanding of their risk for type 1 diabetes from ages 10 to 15 years and to identify factors associated with their risk perception."	Genetically predisposed children, aged 10-15 years	n=4,956	12% FDR, 88% no FDR	TEDDY - Antibody surveillance	No	Annually from aged 10 to 15 years		Children's risk perception			
Quinn *et al.* 2024 (74)	UK	Qualitative (Semi-structured interviews)	Qualitative (Semi-structured interviews, thematic analysis)	*"We undertook a qualitative exploration of the perceptions and perspectives of parents on the proposed screening programme."*	Mothers and Fathers, Children, General Population	n=38 parents	26% FDR T1D, 45% no family history	Hypothetical / attitudinal							
Roth *et al.* 2015 (42)	Europe and US	Uncontrolled cohort	Questionnaire	*"To understand the association between life stress, postpartum depression, maternal perception of her child's risk for type 1 diabetes (T1D), and a mother's anxiety about her child's T1D risk in mothers of genetically at risk children."*	Mothers and Fathers, Genetically predisposed child	n=7612, completed TEDDY visit by 6months	11% FDR T1D and 89% no family history of T1D	TEDDY - Antibody surveillance	No	Post - 6m	SAI, EPDS	Risk perception of child developing T1D			
Scudder *et al.* 2024 (75)	UK	Qualitative (Semi-structured interviews)	Qualitative (Semi-structured interviews, thematic analysis)	*"To assess acceptability from parents whose children were screened and feedback from non-responders."*	Mothers, General Population	n=15 Mothers (interviewed, 49% response rate) and n=32 anonymous postcards (n=29 participated and n=3 did not participate)	95% had no family history of T1D	T1Early - antibody screening	No	Post - not stated	Emotional reaction				Reasons for participation, Screening preferences, Child's response to screening

Simonen *et al.* 2006 (34)	Finland	Cross-sectional, telephone questionnaire	Questionnaire (telephone)	*"To explore the impact of screening, and assess the anxiety, psychosocial reactions, and coping behaviours of parents."*	Mothers and Fathers, General Population	n=443 high risk and n=506 low risk completed questionnaire	NR	DPP - newborn genetic screening (cord blood) and antibody surveillance	No	Post- within 1m (1)	STAI-20, Stress theory-15		Ways of coping checklist-8	Social Readjustment Scale (SRS)	
Sims *et al.* 2019 (76)	International - Australia, Canada, Europe, New Zealand, US	Cross-sectional	Quantitative	*"To better understand potential facilitators of individual engagement in type 1 diabetes natural history and prevention studies through analysis of enrolment data in the TrialNet Pathway to Prevention (PTP) study."*	Parent of antibody positive child	n=2401 aged <18 years	100% family history T1D	Trialnet - Antibody surveillance	No	Cross-sectional, 2017					Factors associated with venous confirmation and OGTT
Smith *et al.* 2014 (48)	Europe and US	Uncontrolled cohort	Questionnaire	*"The current study documents maternal reports of actions to prevent type 1 diabetes during the first year of the TEDDY study. This study also examined the characteristics of mothers who reported such preventive actions, including both sociodemographic and psychological variables."*	Mothers	n=7613 responses (6m), n=6503 (15m), n=6303 completed both (6m and 15m)	NR	TEDDY	No	Uncontrolled cohort			Behavioural modifications and regression analysis		

Smith *et al.* 2018 (43)	Europe and US	Uncontrolled cohort	Questionnaire	*"This study examines the psychological impact of type 1 diabetes in the year following diagnosis on families participating in a long-term observational study compared to families diagnosed in the community."*	Mothers and Fathers, genetically predisposed children	n=54 TEDDY cases with matched community controls	6-item SAI	TEDDY – Antibody surveillance	Yes, matched community controls	3, 6 and 12 month visits in the year after stage 3 T1D diagnosis	6-item SAI, 42-item Paediatric Inventory for Parents, Child diabetes-specific quality of life				
Smith *et al.* 2021 (44)	Europe and US	Uncontrolled cohort	Questionnaire and Clinical research file	*"We examined parental diabetes monitoring behaviors in a cohort of children at increased genetic risk for type 1 diabetes."*	Mothers and Fathers, Genetically predisposed child	n=7319 enrolled after first year (1) n=5944 Ab negative children (n=5929 Mothers and n=5629 Fathers), (2) n=867 parents with IA+ child (n=839 Mothers and n=704 Fathers) and (3) n=777 IA+ children with up to 4 years follow-up (n=771 Mothers and n=712 Fathers)	NR	TEDDY - Antibody surveillance	No	Post - within 7 years			Long-term impact of antibody results on parental monitoring behaviours		
Stolt *et al.* 2002 (77)	Sweden	Qualitative (Semi-structured interviews)	Qualitative (Semi-structured interviews, theoretical bioethical framework)	*"The objective of this paper is to explore and describe ethically relevant opinions and attitudes among a sample of participants and nonparticipants in respect of the ABIS project."*	Mothers, genetically predisposed child	n=21 (n=15 participated in ABIS, n=6 did not participate in ABIS)	NR	ABIS - newborn screening (not routinely informed of result)	No	Post, within 6 months post-partum					General attitudes towards screening research, views on decision making and surrogate decision making, views on information, data collection and storage, views on discovery of high risk

Stolt *et al.* 2003 (78)	Sweden	Cross-sectional, questionnaire	Questionnaire	*"To describe the attitudes and opinions of a representative sample of the participants regarding their involvement."*	Mothers, General population	n=293 (73% response rate)	NR	ABIS - newborn screening (not routinely informed of result)	No	Post - within 6 years					Attitudes towards ABIS study, reasons for participation, attitudes towards research material, right to be informed of a high risk result
Stolt *et al.* 2005 (84)	Sweden	Cross-sectional, questionnaire	Questionnaire	*"To improve our understanding of how participants perceive information, the informed consent process and the aims and methods of the ongoing ABIS study."*	Mothers, General population	n=293 (73% response rate)	Anonymous	ABIS - newborn screening (not routinely informed of result)	No	Post - within 6 years					Satisfaction with and understanding of information provided, informed consent, general aims and methods, individual participation
Swartling *et al.* 2007 (87)	Sweden	Cross-sectional, questionnaire	Questionnaire	*"To assess the preconditions of disclosure."*	Mothers and Fathers, General Population	n=7206 parents	6.3-9.7% had family history of T1D, 23.2-26.5% had family history of T1D/T2D	ABIS - newborn screening (not routinely informed of result)	No	Post - 5 years					Whether parents wished to know child's risk status and reasons for or against
Swartling *et al.* 2008. Parental (79)	Sweden	Cross-sectional, questionnaire	Questionnaire	*"To explore parents' views on issues relating to information, consent and research data."*	Mothers and Fathers, General Population	n=1302 (52% response rate)	NR	ABIS - newborn screening (not routinely informed of result)	No	Post - 7-9 years					Perceptions of confidentiality, informed consent and responsibilities regarding child's participation in research, data management and child's right to assent/dissent

Swartling *et al.* 2008. Self-assessed (86)	Sweden	Cross-sectional, questionnaire	Questionnaire	*"We present data concerning knowledge and self- assessed understanding."*	Mothers and Fathers, General Population	n=5262 parents	8.8% family history of T1D, 26.3% family history of T1D/T2D	ABIS - newborn screening (not routinely informed of result)	No	Post - 5 years		Self-reported level of understanding, knowledge of the aims of the ABIS study			
Swartling *et al.* 2009 (85)	Sweden	Cross-sectional, questionnaire	Questionnaire (mailed)	*"To explore parental views on children's participation in research (and to explore arguments for the viewpoints given) we incorporated questions about children's participation, information and consent, including parents' views on children's right to decide."*	Parents	n=3959	10% FDR	ABIS - newborn screening (not routinely informed of result)	No	Post- 1, 2.5 and 5 years					Views on informing the child, views on child's decision making, parents' views on important values in paediatric research
Swartling *et al.* 2011 (81)	Sweden	Qualitative (focus groups)	Qualitative (focus groups)	*"To explore children's views about medical research and participation."*	Children aged 10-12 years	n=6 focus groups, n=6-8 children FG	Anonymous	ABIS - post follow-up	Pre-ABIS follow-up	2009-2010					Knowledge about research, a sense of altruism, shared decision-making and right to dissent, notions of integrity, privacy, and access, and understanding of disease risk and personal responsibilities.
Swartling *et al.* 2014 (80)	Sweden	Cross-sectional, questionnaire	Questionnaire	*"To explore 10- to 13-year-old boys' and girls' views on medical research, trust, information, decision making, and views on data sampling and risk identification, in the context of long-term screening for type 1 diabetes."*	Children aged 10-14 years	n=5851 children (72% participated ABIS, 28% not participated)	6.7% had a Mother with T1D and 2.9% had T1D	ABIS - newborn screening (not routinely informed of result)	Children who had not participated in ABIS or prior to ABIS participation	2009-2011	Psychological impact				Views on paediatric research, views on information and decision making,

Swartling *et al.* 2016 (47)	US and Europe (Finland, Sweden, Germany)	Uncontrolled cohort	Questionnaire and Clinical research file	*"To assess mothers' and fathers' perception of their child's risk of getting type 1 diabetes (T1D)."*	Mothers and Fathers, Genetically predisposed child	n=432 parents at staging (76% response rate), n=314 at 6 months (61% response rate), and n=256 at 12 months (58% response rate)	11% FDR, 89% no FDR	TEDDY - Genetic and Antibody surveillance	No	Post- within 3m, 6m, 15m and 27m		Parent perception of child's risk, belief that T1D risk can be reduced			
Van Esch *et al.* 2010 (82)	Netherlands	Qualitative (Mailbox questions, secondary analysis	Qualitative (Mailbox questions, secondary content analysis)	*"Our research interest is in developing a profile of questioners, as well as the discovery of themes and tendencies in the emailed questions."*	Diabetes patients, partners and relatives	n=172 emailed questions	49% diabetes patients, 30% relatives, 21% partners	Hypothetical / attitudinal							
Wentworth *et al.* 2025 (83)	Australia	Cross-sectional	Quantitative	*"We describe screening, monitoring, and clinical outcomes over the subsequent 2 years and characteristics and outcomes of people who collected a blood spot at home are compared with those who underwent venipuncture at a community collection center."*	Children aged >2 years	n=793	99% Family history	Targeted (FHx) autoantibody screening	No	Analysis performed after two years of running the trial					Participant choice for screening method and return rates

Yu *et al.* 1999 (39)	US	Uncontrolled time series - questionnaire	Questionnaire (mailed)	*"The purpose of the study was to evaluate the impact of receipt of information regarding genetic risk of type 1 diabetes in the newborn on maternal stress level and 2) to evaluate whether maternal variables such asage, race, marital status, education level, family in-come, or child's birth order were predictive of maternal parenting stress response."*	Mothers, General population	High vs Low risk (total n=88): High risk (n=23) (included high (n=3) and intermediate (n=20)), low risk (n=65) (paired questionnaire completers)	3.4% Mothers had a family history of diabetes, 0% of children had a relative with diabetes	DAISY - genetic newborn screening	No	Pre - 5-7 weeks post-partum and Post - within 6m (2)	Parenting Stress Index (PSI-36) and Total Stress Score (TSS)				
Ziegler *et al.* 2020 (4)	Germany	Uncontrolled time series - questionnaire	Questionnaire	*"The Fr1da study assessed the prevalence of presymptomatic type 1 diabetes in children participating in a public health screening program for islet autoantibodies, the risk for progression to clinical diabetes and diabetic ketoacidosis, and parental psychological stress."*	Mothers and Fathers, General Population	n=432 parents at staging (76% response rate), n=314 at 6 months (61% response rate), and n=256 at 12 months (58% response rate)	11% FDR, 89% no FDR	Fr1da - antibody screening and surveillance	DiMelli T1D diagnosed cohort	Post - immediately post notification, within 6m and 12m	Patient Health Questionnaire (PHQ-9)				

ESM Table 2 Legend

Characteristics of each of the 70 included studies describing the aims, design, population, assessments and outcomes. Abbreviations: NR – not reported, SDR – second degree relative, GDM – Gestational diabetes. References: 4, 13-14, 24-87, 99-101.

ESM Table 3 - Excluded articles and reasons for exclusion

Author	Year	Title	Reason for exclusion
Anonymous	2005	*Program: Genetic Information for Testing Type 1 Diabetes (GIFT-D)*	Cannot access full text from three institutions
Baughcum	2005	*Maternal efforts to prevent Type 1 diabetes in genetically screened infants.*	Dissertation
Bingley	2015	*Use of Dried Capillary Blood Sampling for Islet Autoantibody Screening in Relatives: A Feasibility Study*	Wrong population - cannot discern paediatric from adult data
Carel	1992	*Screening of type I diabetes in patients' families*	Wrong outcomes - No implications or acceptability assessment
Carmichael	2004	*Newborn genetic screening for type 1 diabetes: Factors affecting maternal risk perception, anxiety and study participation.*	Dissertation
Galatzer	2001	*Psychological impact of islet cell antibody screening*	Cannot access full text from three institutions
Gesualdo	2012	*Effectiveness of an Informational Video Method to Improve Enrolment and Retention of a Pediatric Cohort*	Wrong study design - Intervention to increase retention
Gesualdo	2024	Structures and Strategies for Retaining an International Pediatric Cohort from Birth: Lessons from The Environmental Determinants of Diabetes in the Young (TEDDY) Study	Wrong study design - Intervention to increase retention
Giannattasio	2010	*Estimation of genetic risk for Type 1 diabetes mellitus in newborns on dried blood spot.*	Wrong outcomes - No implications or acceptability assessment
Goldstein	2009	*From genetic risk awareness to overt type 1 diabetes: parental stress in a placebo-controlled prevention trial.*	Wrong study design and outcomes - Screening for entry to an immunoprevention trial
Hibi	1978	*Screening diabetes mellitus in children--its problems and future*	Cannot access full text from three institutions
Hood	2004	*Maternal response to newborn genetic screening for type 1 diabetes: The role of depression.*	Dissertation
Ilonen	1996	*Population-based genetic screening for IDDM susceptibility as a source of HLA-genotyped control subjects*	Wrong study design - Narrative review with no original data
Johnson	2001	*Screening programs to identify children at risk for diabetes mellitus: Psychological impact on children and parents*	Wrong study design - Letter to the editor with no original data
Johnson	2014	*At high risk for early withdrawal: using a cumulative risk model to increase retention in the first year of the TEDDY study.*	Wrong study design - Intervention to increase retention
Kireeva	1971	*Mass screening for diabetes mellitus with carbohydrate loading using the preparation "Glucotest"].*	Cannot access full text from three institutions
Kitagwa	1979	*Mass screening of diabetes mellitus*	Cannot access full text from three institutions
Knopf	1977	*Oral glucose tolerance test in 100 normal children*	Wrong outcomes - No implications or acceptability assessment

Kordonouri	2019	*Fr1dolin: Pediatric population screening for type 1 diabetes (T1D) and familial hypercholesterolemia (FH) in Lower Saxony, Germany*	Wrong outcomes - No implications or acceptability assessment
Kordonouri	2022	*Type 1 diabetes risk screening in newborns and studies on the prevention of type 1 diabetes: Better understanding of interactions and tailored approaches for prevention*	Cannot access full text from three institutions
Lorini	2005	*Screening for type 1 diabetes genetic risk in newborns of continental Italy. Primary prevention (Prevefin Italy)--preliminary data.*	Wrong outcomes - No implications or acceptability assessment
Lucidarme	2008	*Appraisal and implications of predictive testing for insulin-dependent diabetes mellitus*	Cannot access full text from three institutions
Ludvigsson	2001	*Screening for pre-diabetes in the general child population may be reassuring.*	Cannot access full text from three institutions
Michalkova	1978	*Results of screening for early developmental stages in juvenile diabetes mellitus].*	Cannot access full text from three institutions
Nwose	2017	*Development of public health program for type 1 diabetes in a university community: preliminary evaluation of behavioural change wheel.*	Wrong population - cannot discern parents' views from adult views
Puff	2016	*Early diagnosis, early care--"Fr1da" screening of children for type 1 diabetes*	Wrong study design - Narrative review with no original data
Raab	2016	*Capillary blood islet autoantibody screening for identifying pre-type 1 diabetes in the general population: Design and initial results of the Fr1da study*	Wrong outcomes - No implications or acceptability assessment
Rains	2019	*A test of the risk perception attitude framework as a message tailoring strategy to promote diabetes screening.*	Wrong population - adults with T2D
Roberts	2017	*Receptiveness to participation in genetic research: A pilot study comparing views of people with depression, diabetes, or no illness.*	Wrong population - adult views
Sims	2022	*Screening for Type 1 Diabetes in the General Population: A Status Report and Perspective*	Wrong study design - Narrative review with no original data
Simmons	2019	*Screening children for type 1 diabetes-associated antibodies at community health fairs.*	Wrong outcomes - No implications or acceptability assessment
Sing	2024	*Feasibility and Validity of In-Home Self-Collected Capillary Blood Spot Screening for Type 1 Diabetes Risk*	Wrong population - cannot discern paediatric from adult data
Stolt	2002	*Parents want to know if their child is at high risk of getting diabetes*	Low quality (methods/results)
Vialettes	1992	*Should screening for Type 1 diabetes in offspring of insulin dependent parents be encouraged?*	Wrong study design - Narrative review with no original data
Wadling	2023	*Type 1 Diabetes National Screening Pilot: Feasibility and Acceptability Study*	Cannot access full text from three institutions
Ziegler	2019	*Screening for asymptomatic beta-cell autoimmunity in young children.*	Wrong outcomes - No implications or acceptability assessment

ESM Table 3 Legend
Excluded articles and reasons for exclusion.

ESM Table 4a and b – Quality assessments

ESM Table 4a – Quality assessment of quantitative articles

Article (reference)	Quantitative					MMAT score	Proportion (Relative to the total number of criteria)	MMAT grade	CASP grade
	**1.1. Is the sampling strategy relevant to address the research question? *(Are participants approached appropriate?)***	**1.2. Is the sample representative of the target population? *(Representative of general population, responders vs non-responders/ representative of screened population if stated)***	**1.3. Was a validated measurement/ tool used to assess psychosocial impact?**	**1.4. Is the risk of nonresponse bias low? *(Proportion returned questionnaires)***	**1.5. Is the statistical analysis appropriate to answer the research question? *(descriptive p<0.05, stated statistical tests (parametric or non-parametric, regression analysis)***				
Aas *et al.* 2010 (36)	Y	N	N	Y	N	2	0.4	low	moderate
Angiulli *et al.* 2025 (52)	Y	CT	N	Y	Y	3	0.6	moderate	moderate
Baughcum *et al.* 2005 (50)	Y	CT	Y*	N	Y	3	0.6	moderate	moderate
Baxter *et al.* 2012 (53)	Y	CT	N	Y	Y	3	0.6	moderate	high
Bendor-Samuel *et al.* 2023 (54)	CT	CT	N	CT	CT	0	0	low	low
Carmichael *et al.* 2003 (35)	Y	N	Y*	CT	Y	3	0.6	moderate	moderate
Driscoll *et al.* 2021 (55)	Y	CT	Y*	Y	Y	4	0.8	moderate	moderate
Dunne *et al.* 2021 (56)	Y	Y	N	Y	Y	4	0.8	moderate	moderate
Galatzer *et al.* 2001 (99)	Y	CT	N	N	Y	2	0.4	low	moderate
Gesualdo *et al.* 2016 (58)	Y	CT	N	N	Y	2	0.4	low	moderate
Helgesson *et al.* 2008 (59)	Y	CT	N	CT	CT	1	0.2	low	moderate
Helgesson *et al.* 2009 (60)	Y	CT	N	N	CT	1	0.2	low	moderate
Hendrieckx *et al.* 2002 (49)	Y	CT	N	Y	Y	3	0.6	moderate	moderate

Hommel *et al.* 2018 (100)	Y	Y	N	CT	Y	3	0.6	moderate	moderate
Hood *et al.* 2005 (40)	Y	N	N	CT	Y	2	0.4	low	moderate
Hood *et al.* 2006 (45)	Y	N	Y*	CT	Y	3	0.6	moderate	high
Houben *et al.* 2022 (41)	Y	Y	N	CT	Y	3	0.6	moderate	moderate
Hummel *et al.* 2004 (26)	Y	CT	Y*	Y	Y	4	0.8	moderate	moderate
Johnson *et al.* 1990 (27)	Y	CT	Y*	Y	CT	3	0.6	moderate	moderate
Johnson *et al.* 1995 (28)	Y	CT	Y*	Y	Y	4	0.8	moderate	moderate
Johnson *et al.* 2000 (101)	Y	CT	Y*	Y	Y	4	0.8	moderate	moderate
Johnson *et al.* 2004 (29)	Y	N	Y*	CT	Y	3	0.6	moderate	moderate
Johnson *et al.* 2011 (30)	Y	CT	N	Y	Y	3	0.6	moderate	high
Johnson *et al.* 2016 (61)	Y	CT	N	Y	Y	3	0.6	moderate	high
Johnson *et al.* 2017 (31)	Y	Y	Y*	Y	Y	5	1	high	high
Kelly *et al.* 2024 (62)	Y	CT	N	CT	Y	2	0.4	low	moderate
Kerruish *et al.* 2007 (14)	Y	Y	Y*	Y	Y	5	1	high	moderate
Kerruish *et al.* 2017 (32)	Y	CT	N	Y	Y	3	0.6	moderate	high
Kick *et al.* 2019 (51)	Y	CT	N	N	Y	2	0.4	low	low
Kupila *et al.* 2001 (63)	Y	Y	N	Y	Y	4	0.8	moderate	moderate
Lernmark *et al.* 2004 (37)	Y	Y	N	Y	Y	4	0.8	moderate	moderate
Lernmark *et al.* 2011 (64)	Y	Y	N	Y	Y	4	0.8	moderate	high
Lernmark *et al.* 2012 (65)	Y	CT	N	N	Y	2	0.4	low	moderate
Lernmark *et al.* 2016 (66)	Y	CT	N	Y	Y	3	0.6	moderate	high
Liu *et al.* 2017 (67)	Y	CT	N	CT	Y	2	0.4	low	moderate
Ludvigsson *et al.* 2001 (38)	Y	Y	N	Y	CT	3	0.6	moderate	moderate
Melin *et al.* 2020 (33)	Y	CT	Y*	CT	Y	3	0.6	moderate	high
Melin *et al.* 2022 (71)	Y	CT	Y*	CT	Y	3	0.6	moderate	moderate

Melin *et al.* 2023 (70)	Y	CT	N	Y	Y	3	0.6	moderate	high
O'Donnell *et al.* 2023 (13)	Y	Y	Y*	Y	Y	5	1	high	high
O'Donnell *et al.* 2025 (46)	Y	Y	N	CT	Y	3	0.6	moderate	moderate
Roth *et al.* 2015 (42)	Y	Y	Y*	Y	Y	5	1	high	high
Simonen *et al.* 2006 (34)	Y	N	Y*	N	Y	3	0.6	moderate	moderate
Sims *et al.* 2019 (76)	Y	CT	N	Y	Y	3	0.6	moderate	high
Smith *et al.* 2014 (48)	Y	Y	N	Y	Y	4	0.8	moderate	high
Smith *et al.* 2018 (43)	Y	CT	Y*	CT	Y	3	0.6	moderate	moderate
Smith *et al.* 2021 (44)	Y	CT	N	Y	Y	3	0.6	moderate	moderate
Stolt *et al.* 2003 (78)	Y	CT	N	Y	CT	2	0.4	low	moderate
Stolt *et al.* 2005 (84)	Y	CT	N	Y	CT	2	0.4	low	low
Swartling *et al.* 2007 (87)	Y	CT	N	CT	Y	2	0.4	low	moderate
Swartling *et al.* 2008._Sef-assessed (86)	Y	CT	N	CT	Y	2	0.4	low	moderate
Swartling *et al.* 2008. Parental (79)	CT	CT	N	CT	Y	1	0.2	low	low
Swartling *et al.* 2009 (85)	Y	N	N	CT	Y	2	0.4	low	moderate
Swartling *et al.* 2014 (80)	Y	CT	N	CT	CT	1	0.2	low	low
Swartling *et al.* 2016 (47)	Y	CT	N	Y	Y	3	0.6	moderate	moderate
Wentworth *et al.* 2025 (83)	Y	CT	N	N	Y	2	0.4	low	moderate
Yu *et al.* 1999 (39)	Y	Y	N	N	Y	3	0.6	moderate	high
Ziegler *et al.* 2020 (4)	Y	Y	N	Y	Y	4	0.8	moderate	high

ESM Table 4b – Quality assessment of qualitative articles

Article (reference)	Qualitative					MMAT score	Proportion (Relative to the total number of criteria)	MMAT grade	CASP grade
	1.1 is the qualitiative approach appropriate for the research question?	1.2 Are the qualitative data collection methods adequate to address the research question?	1.3 Are the findings adequately derived from the data?	1.4 Is the interpretation of results sufficiently substantiated	1.5 Is there coherence between qualitative data sources, collection, analysis and interpretation				
Faustini *et al.* 2025 (57)	Y	Y	Y	N	Y	4	0.8	moderate	moderate
Kerruish *et al.* 2011 (25)	Y	Y	Y	Y	Y	5	1	high	moderate
Kerruish *et al.* 2016 (24)	Y	Y	Y	Y	Y	5	1	high	moderate
Litchfield *et al.* 2024 (68)	Y	Y	Y	N	Y	4	0.8	moderate	moderate
Ludvigsson *et al.* 2002 (69)	Y	Y	Y	N	N	3	0.6	moderate	moderate
Nicholls *et al.* 2013 (72)	Y	Y	Y	Y	N	4	0.8	moderate	moderate
Nicholls *et al.* 2016 (73)	Y	Y	N	N	N	2	0.4	low	low
Quinn *et al.* 2024 (74)	Y	Y	Y	N	Y	4	0.8	moderate	moderate
Scudder *et al.* 2024 (75)	Y	Y	Y	N	N	3	0.6	moderate	moderate
Stolt *et al.* 2002 (77)	Y	Y	Y	Y	N	4	0.8	moderate	moderate
Swartling *et al.* 2011 (81)	Y	Y	Y	Y	Y	5	1	high	moderate
Van Esch *et al.* 2010 (82)	N	Y	Y	Y	Y	4	0.8	moderate	moderate

ESM Table 4a and b Legend

Quality assessment for each of the 70 included studies using the MMAT (mixed methods appraisal tool). Abbreviations: Y – yes (green), N – No (red), CT – cannot tell (amber), MMAT – Mixed Methods Appraisal Tool, CASP – Critical Appraisal Skills checklist. We used a modified question 1.3 of the MMAT: “Was a validated measurement/tool used to assess psychosocial impact?” instead of “was an appropriate measure used?”

Key: *The state trait anxiety and state anxiety inventory (SAI, STAI-10 and STAI-Children) assess state anxiety and are validated to assess parental diabetes-specific anxiety when participating in a screening/early detection programme. References: 102-106.

ESM Table 6 – Positive and negative indicators of acceptability

Article (reference)	Intervention / Study	Population	Positive indicators of acceptability	Negative indicators of acceptability
Angiulli *et al.*2025 (52)	UNI SCREEN	Mothers, Fathers. Children, General population	Pre-post screening: agree and strongly agree all very high acceptability and in all but one increased after screening: (99.6%-99.6%) I believe this screening program will be useful for paediatric population to prevent future health problems.<br>(98.4-100%) I believe this screening program will be useful for my child to prevent future health problems.<br>(98,8-98,8%) I support the idea of population screening for diabetes, celiac disease and cardiovascular disease.<br>(94.2-91.7%) I believe this program will improve my child's quality of life.<br>(85.5-89.6%) I believe this program will help my child change his/her lifestyle.<br>(96.7- 99.2%) I find this program safe in terms of health effects and data confidentiality.<br>(85.9-97.5%) I have understood what my child would have to do in case of a positive screening test result.<br>(99.2-100%) I understood the purpose of the program.<br>As for feasibility assessed post screening:<br>(98.8%) I am satisfied with the service my child received. (100%) All information about the program was clear and easy to understand.<br>(99.6%) I would recommend this screening program.<br>The response rate was over 50% for screening; this was also confirmed by the high response rate when confirmatory testing was required, with venous blood sampling showing less than 5% of participants were lost to follow-up.	

Baxter *et al.* 2012 (53)	TEDDY - Genetic and Antibody surveillance	Mothers and Fathers, Genetically predisposed child	Enrolment rates were higher among FDR than GP participants and among families with another child already enrolled regardless of ethnic minority status.<br><br>Reasons for continuation (enrolment to follow-up): ethnic differences in enrolment were minor (only other minority participants had substantially lower enrolment rates than non-Hispanic white. Predictors: Of those who enrolled, higher proportion of mothers who chose to enrol their child in TEDDY had 12 years or more education compared to those who refused/were excluded for both non-Hispanic white and Hispanic groups.<br><br>Older Mothers and FDR were less likely to be excluded.	Reasons for non-participation: Primary reasons for not enrolling were demands of the study protocol and family considerations. African American and other minority were more likely not to provide a reason for refusing enrolment, Other minority more likely to list moving and wanting to wait and see as reasons for refusing. Predictors on regression analysis: Other minority were significantly less likely to enrol, Washington clinic participants less likely to enrol, and being a twin was associated with not enrolling.<br><br>Reasons for withdrawal: Most common reason for exclusion was no response to calls or messages. Predictors of exclusion: proportion of HLA eligible children excluded was significantly higher amongst minority groups (twice as common) i.e. Hispanic, other minority and non-Hispanic white. Participants in Georgia and Florida were more likely to be excluded and Colorado centre less likely to be excluded. Predictors of exclusion: 41% of those excluded had <12 years education compared with 22% of those who were not excluded.<br><br>Reasons for exclusion/discontinuation: Ethnic minorities with lower educational level were more likely to be excluded than non-Hispanic white with lower educational level.<br><br>Early withdrawal was higher in ethnic minorities compared to non-Hispanic white, primary reasons: 1) blood draw, 2) frequency of visits, 3) stool sample collection, 4) being too busy, 5) passive withdrawal.<br>Predictors of early withdrawal: Hispanic ethnicity was a significant predictor of early withdrawal as was being a younger mother, in addition to mothers who smoked during pregnancy and/or never drank alcohol. Mothers who underestimated their child's risk were more likely to withdraw, and families with a female TEDDY child were more likely to withdraw, and among mothers with accurate risk perceptions, those with high anxiety were more likely to withdraw.
Bendor-Samuel *et al.* 2023 (54)	INGR1D + POINT - GPPAD	Mothers, General population	Reason for participation: absence of additional intervention.	Reasons for non-participation: 1) data protection concerns, 2) fear of entire genome sequencing and potential exploitation of genetic data, 3) test accuracy i.e. sensitivity level at 25%, women worried about the value of a negative screening result and with a predictive value of 10%, this may lead to unwarranted worry, 4) felt screening was not relevant because they had no family history of T1D and assumed child would be low risk.<br><br>Reasons for withdrawal: 0.68% withdrew due to 1) technical error (sampling), 2) issues with consent or withdrew consent, 3) moving/out of area.
Driscoll *et al.* 2021 (55)	TEDDY - Genetic and Antibody surveillance	Mothers, Genetically predisposed child	Reasons for continuation: OGTT adherence - those who were most adherent to OGTT protocol included 1) mothers who perceived their child is at increased risk of T1D, 2) those who reported monitoring the child for T1D or glucose monitoring at home, 3) greater maternal satisfaction with TEDDY, 4) Finnish and older participants from Sweden were more likely to adhere than US.	Lower adherence to study continuation observed in 1) first born children, 2) FDR with T1D, 3) being on long distance protocol and 4) those who underestimated their child's risk of T1D.
Dunne *et al.* 2021 (56)	Hypothetical / attitudinal	Mothers and Fathers, Children, General Population	Attitudes and screening preferences (hypothetical): parents preferred screening and monitoring programmes that 1) could reduce DKA risk to 1% per year, 2) with a treatment to delay stage 3 T1D onset and 3) avoid an out of pocket cost ($50). Screening test mode of administration, screening location and timing of the screening were relatively less important to parents.	

Faustini *et al.* 2025 (57)	Hypothetical / attitudinal	Mothers and Fathers, Children, General Population	Screening preferences: Both parents and stakeholders thought DBS sampling offered a minimally invasive screening test, which could be most conveniently performed at home (Theme: Convenience and ease of DBS testing). Known advantages of DBS home-testing include reduced geographical and social barriers for parents, i.e. less time off work or school and less distress for children from sampling in a familiar, non-clinical setting. Although parents recognise short-lived discomfort with finger-prick sampling, home-testing was still preferred over serum collection (Theme: Finger-prick testing experience increased parent's confidence in home-testing). All concerns raised by parents regarding home-testing were resolved if DBS testing was offered by a HCP in a community setting (Theme: Community DBS testing to improve accessibility and uptake).	Parents recognised short-lived discomfort with finger-prick sampling.<br>Concerns raised by the parent cohort included lack of experience with finger-prick testing, difficulty restraining a young child, incorrect test completion, obtaining insufficient sample, sanitary issues and safeguarding concerns.
Gesualdo *et al.* 2016 (58)	Antibody screening (paediatric centre)	Mothers and Fathers, General Population	Screening experience: 1) Staff assessed child's reaction to the blood draw and found that 79% of children had a great reaction, 11% good reaction, 7.5% neutral, 1.5% bad and 0.5% very bad, similar by age and sex. 2) Parents were satisfied with all aspects of the study with no difference between mothers or fathers, 3) parents appreciated the value of the research staffs' knowledge and experience, 4) parents selected finger stick more than venepuncture, as mean blood volume was higher from venepuncture, 5) parental satisfaction did not differ by blood collection method (finger stick or venepuncture).	
Helgesson *et al.* 2008 (59)	ABIS - newborn screening (not routinely informed of result)	Mothers and Fathers, General Population	Reasons for participation: 1) 76% said it may aid their own child, 2) 78% said it may aid other children, 3) 77% said it may aid useful research, 4) 1.1% stated "other".	Reasons for non-participation: 1) 0.4% expressed concern about participation (scoring 1-3 on the Likert scale) – no further information provided.
Helgesson *et al.* 2009 (60)	ABIS - newborn screening (not routinely informed of result)	Mothers and Fathers, General Population	Reasons for participation: 1) 97% said benefits of research, 2) 83% said identification that child is high risk, 3) 81% said they trusted the researchers, 4) 68% said because the child would be medically examined, 5) 63% said there were advantages of participating for their child or themselves, 6) parents with lower educational level stressed diseases in near or extended family as an important factor for participation and that the child would be medically examined.	Reasons for non-participation: 1) 39% said lack of time, 2) 36% said blood sampling procedures, 3) 21% said identification of children being at high risk of T1D, 4) 4% language difficulties, 5) 8% had plans to move out of area, 6) 10% lacked trust in the researchers, 7) 5% gave other reasons e.g. other diseases as a reason for non-participation , 8) younger mothers quoted lack of information as a reason for opting out, 9) fathers with lowest educational level reported high risk identification as reason for opting out.<br><br>Reasons for discontinuation: 1) 21% had no reported experience of ABIS (of these 32% said they had never been asked to participate in ABIS), 2) 53% said lack of time, 3) 42% said sampling procedures, 4) 34% said lack of information, 5) 7% said they could no longer trust researchers, 6) parents with lower educational level stated more frequently that storage and analysis of blood samples were important to their decision to dropout, and 7) concerns that the child would be medically examined, 8) dropouts were significantly more dissatisfied with the general study information, wishing more in depth information about the general purpose, aims and procedures of the ABIS study were provided.

Johnson *et al.* 1990 (27)	TEDDY - Antibody surveillance	Mothers and Fathers, Genetically predisposed child	Screening experience: At follow-up, all children had a positive reaction towards study participation.	Screening experience: Initially, antibody positive children wished they had not participated, because the blood taking hurt.
Johnson *et al.* 1995 (28)	Antibody screening	ICA+ children and at least one parent (excluded ICA+ adult dataset)		Screening experience: 10% of antibody positive children wished they had not participated citing concerns about the pain associated with blood draws.
Johnson *et al.* 2011 (30)	TEDDY - Antibody surveillance	Mothers and Fathers, Genetically predisposed child		Reasons for discontinuation: Predictors of early withdrawal included 1) US and German sites higher than Sweden or Finland sites, 2) younger maternal age, 3) mothers who smoked during pregnancy or never drinking alcohol in pregnancy was associated with higher withdrawal rate, 4) reducing working hours, quitting or not working at all during pregnancy was associated with higher withdrawal rates, 5) 43% of families with no paternal participation withdrew from the study in the first year compared to 18% of families with paternal involvement (completing questionnaire), 6) of mothers with more accurate risk perception, the more anxious were more likely to withdraw, 7) in total 91% had complete data for analysis but of those with >1 missing data point, 58% withdrew in first year compared to only 19% who withdrew with one or fewer missing data points.
Johnson *et al.* 2016 (61)	TEDDY - Antibody surveillance	Mothers and Fathers, Genetically predisposed child		Reasons for discontinuation: Predictors of withdrawal included the following demographic factors, 1) only child status, 2) younger maternal age, 3) lower maternal educational level and 4) household crowding; the following maternal lifestyle behaviours, 1) not working or reducing hours during pregnancy and 2) smoking (during pregnancy); the following maternal reactions, 1) maternal risk perception accuracy underestimated risk were more likely to leave; In study behaviour, 1) participants recruited in the earliest years, 2) fathers who did not complete surveys and 3) those who missed blood draws or study visits and 4) mothers least satisfied with TEDDY more likely to withdraw.
Kelly *et al.* 2024 (62)	Other - Antibody screening or hypothetical	Mothers and Fathers, FHx T1D	Screening experience: 70.8% positive, 23.6% neutral and 5.6% somewhat negative and 97% would decide to screen their child again.	
Kerruish *et al.* 2016 (24)	KEA - cord blood genetic risk and antibody surveillance	Mothers and Fathers, General Population	Reasons for participation: Retrospective: What conditions might be included in population testing programmes? 1) there should be something that could be done to prevent it or minimise risk, 2) some parents thought it was only worth testing for serious conditions (not conditions you could live with), 3) others thought testing should only be considered if the test predictive high chance of developing the condition e.g. 1 in 3, 4) whereas others thought it would be worth including conditions whereby your chances might be quite small but you could die overnight.<br><br>Screening experience: 1) Parents thought there had been no adverse effect. None of the parents said they were worried about their child. Parents were pleased to assist with research but did not view genetic testing process as beneficial, potential for benefit should their child become unwell which they sometimes construed as a very minor benefit, however a few parents did place a little more weight on the benefits of	Screening experience: 1) parents felt the regular follow-up blood tests were difficult , 2) also whilst trying to implement healthy lifestyle strategies (although had not received any specific advice relating to this), this sometimes led to difficulties with extended family members who wanted to give treats to the child.<br><br>Regarding T1D genetic population screening, 1) parents felt there was potential for worry and overprotective behaviour, and 2) this would likely apply to some more than others but it could be very difficult to predetermine this, and 3) timing: felt newborn period was not the right time for testing, and that later in childhood would be preferable, because of the intensity of parenting in the newborn period, effects of tiredness and postpartum hormonal changes on maternal mental state and potential to alter bonding between parent and child, 4) some were sceptical that a genetic test result would assist in the difficult task of changing people's

			simply knowing regardless of whether this information was ultimately going to impact their child's health and wellbeing. 2) Regarding views towards genetic testing in general, parents saw benefits relating to awareness, early detection, and prevention of disease.	behaviour, 5) others thought knowing about a potentially life limiting condition would be too stressful, 6) several parents discussed how difficult it would be to know where to draw the line.
Kick *et al.* 2019 (51)	Fr1da - antibody screening	Mothers and Fathers, General Population	Screening experience: 1) 84% very satisfied or satisfied with the communication of diagnosis of presymptomatic T1D by their primary care paediatrician, 2) education training was also mostly positively judged, 3) majority of parents were satisfied with support received from coordinating centre including opportunity to contact someone with questions, the organisation of the Fr1da study and the written information provided, 4) 80% at 6 months assessed their decision to participate in Fr1da as very good/good.	
Kupila *et al.* 2001 (63)	DIPP - newborn genetic screening and surveillance	Parents, General Population	Occasionally parents who refused the follow-up when they first heard about the risk, joined the trial later.	Reasons for discontinuation / Reasons for withdrawal included 1) worries about the blood tests, 2) long distance or travelling difficulties to the clinic, 3) distress in the family other than the study, 4) lack of time, but 5) half of families gave no reason for withdrawal.

Lernmark *et al.* 2011 (64)	TEDDY - Antibody surveillance	Mothers and Fathers, General Population	Reasons for participation: Participants more likely to enrol in TEDDY were 1) infants born in a European country, 2) older mother, 3) FDR family, and 4) another child already in TEDDY.	Reasons for non-participation: Refused enrolment: 1) TEDDY protocol 38% primary reason for refusal in general population and FHx families, for Finland most common was no reason given 50%, family reasons across all countries 33% among general population (GP) and FHx families, 4% said they were unavailable due to impending moves or other reasons for unavailability, a few parents perceived child's risk not great enough to justify study participation and preferred to wait and see what might happen in the future. Protocol concerns: blood draw 18% (19% GP and 14% fDR) (5% Finland to 36% Germany), demanding nature of study protocol 15%, Sweden>others, travel to the teddy clinic was an obstacle for 9% prominent in Sweden and Finland, some parents reported they did not want to be reminded of child's risk, 1-2% us and Finland vs 5-6% Germany and Sweden, length of the study 3%, family factors: being too busy 34% Sweden vs 5% Germany, Less likely to enrol if child was a twin or triplet.<br><br>Reasons for discontinuation: Reason for exclusion: family did not respond to calls/messages/letters about risk information or to schedule an appointment - passive refusers, 73% of families excluded from teddy, comprised 17% of HLA eligible families and significantly more likely in GP 18% than FHx families 6%, in all countries, passive refusal was most common reason for exclusion, US 27% passive refusers vs 16% Germany, 1% Finnish and 3% Swedish, Second most common reason for exclusion was failure to schedule teddy appointment before child was 4.5months 16% of those excluded, another 8% had incorrect contact information preventing staff contacting the family more of a problem in the US, 24 children excluded because HLA status unknown to schedule appointment prior to 4.5m, 7 were excluded due to a disease or birth defect and 1 child parents refused storage of lab specimens in NIH repository.
Lernmark *et al.* 2012 (65)	TEDDY - Antibody surveillance	Mothers and Fathers, General Population	Screening experience: Parents were very satisfied with TEDDY.<br><br>Reasons for continuation: TEDDY questionnaire completers were more likely to 1) come from a European TEDDY country, 2) higher maternal education, 3) older child, 4) higher study compliance in first year and 5) accurate perception of child's T1D risk.<br><br>Reasons for adherence: 1) having someone watching the child for development of T1D was most commonly selected reason, 2) helping science discover causes of T1D, 3) getting the child's antibody results. 4) In Sweden, also noted being seen by the same TEDDY staff was of greater importance than getting the antibody result. 5) Most parents said they had never thought of leaving TEDDY.	Screening experience: Suggested areas for improvement were as follows 1) Finland sites to focus on day and time of visit scheduling, time needed to complete TEDDY visit, parking; 2) Sweden sites: visit reminders, and parking; 3) US sites: time to complete visit, transportation to the visit and parking.<br>Suggestions for improvement: 1) improve diet records 14%, 2) improve stool sample collection 15%, 3) visit scheduling and reminders 14%, 4) logistics associated with the clinic visit 18%, 5) desire to have more information about TEDDY findings 15%.<br><br>Reasons for discontinuation: Of the parents who had considered discontinuing, reasons were: 1) blood draws 34%, 2) too busy not enough time 18%, 3) protocol too demanding 16%, 4) food diary too demanding 13%, 5) no reason 4%. Predictors of considering leaving TEDDY: 1) German and Finnish participants more than Swedish or US participants, 2) more educated respondents were more likely to consider leaving.

Lernmark *et al.* 2016 (66)	TEDDY - Antibody surveillance	Mothers and Fathers, General Population	Screening experience: concerns about blood draw were associated with mothers' study satisfaction at 6 months and last visit prior to withdrawal.	Screening experience: Parents with lower TEDDY satisfaction were more likely to report concerns with blood draw as a reason for leaving TEDDY.<br><br>Reasons for discontinuing: 1) 27% did not respond to repeated scheduling attempts for >1 year, 2) study withdrawal highest during first year of study and active withdrawal (AW)>passive withdrawal (PW).<br>Predictors: US highest frequency of passive withdrawals, Finland had lowest PW, Germany and Sweden significant increased trend of PW proportion over study period although total number of AW and PW decreased. PW more likely: young mums, older children. AW, young mothers, <25 years and significantly more anxious, and more worried about child developing diabetes. Reasons for AW: 1) protocol: concerns about blood draw 55%, protocol too demanding, transportation difficulties, frequency of visits, concern about blood draw more often mentioned from older children and not wanting to be reminded of child's risk was a significantly more often reported reason during the first visits, 2) family factors: being too busy, not having enough time 67%, feeling overwhelmed/being too stressed 23%, and being too busy/not enough time was more frequently reported in later visits, 11% families moved out of area, 11% did not want to give a reason or wanted to wait and see what might happen. AW predictors: German and US more likely to report blood draw as reason, blood draw more often if older child or girl, being too busy was most often given by Swedish mothers, more common with older child and boys.
Liu *et al.* 2017 (67)	Trialnet - Antibody screening	Children, aged 8-18 years, FDR screening	Screening experience: 1) 90% of those aged ≤8 years and 83% of those aged 9-18 years preferred capillary sampling, significantly greater preference among younger children; 2) ease of testing, from 1-7, median score was 3 in ≤8 years and 3 in aged 9-18 years	

Litchfield *et al.* 2024 (68)	Hypothetical /attitudinal	Mothers, Fathers, children, general population	Screening preferences: Developing communities online and in-person (with peer). Parents suggested that children and young people who have tested positive might be better prepared by facilitated networking with those with established T1D. In this way, they might 'normalise' the associated risk of living with T1D, reduce feelings of isolation and gently introduce them to the management.<br>Integration with Formal Healthcare and Social Support: Parents viewed peer support as an accompaniment to clinical monitoring for children with pre-T1D.	
Ludvigsson *et al.* 2001 (38)	ABIS - newborn screening (not routinely informed of result)	Mothers and Fathers, General Population		Reasons for non-participation: 1) Moving soon, 2) Husband does not agree, 3) Difficult to answer all the questions, 4) Rarely said they were afraid of hurting their child or afraid that their child would not allow capillary blood samples to be taken.

Ludvigsson *et al.* 2002 (69)	ABIS - newborn screening (not routinely informed of result)	Mothers and Fathers, General Population	Reasons for participation: 1) Some saw a possible benefit for their own child (i.e. possibility to prevent diabetes), 2) Majority of mothers believed that such a large study would give important results, 3) Wanting to support research - all interviewed mothers in ABIS were positive towards the project.<br><br>Reasons for non-participation: 1) Those who did not give informed consent were positive about the ABIS study, and 2) One Mother said she was never informed about ABIS.<br><br>Screening experience: Despite some mothers being concerned about the blood tests, the pain was regarded as minimal. Thought blood test was acceptable in relation to the possible benefits of the study from risk notification.	Reasons for non-participation: 1) Two mothers were partly influenced by their spouse, 2) a few mothers were afraid of hurting their child with blood sampling procedures.<br><br>Screening experience: Some mothers were concerned about the blood tests.
Melin *et al.* 2022 (71)	TEDDY – Antibody surveillance	Mothers and Fathers, Genetically predisposed child	Screening experience: Study satisfaction was measured by three items: 1) "Overall, how do you feel about having your child participate in the TEDDY study? (scored 2 =like it a lot, 1 =like it a little, 0 =it is ok or dislike it)," 2) "Do you think your child's participation in TEDDY was a good decision? (scored: 2 = a great decision, 1 = a good decision, 0= an ok decision or bad decision)'" and 3) "Would you recommend the TEDDY study to a friend? (scored: 2 =yes, 1 =maybe, 0 =no)."<br>At child-age 15- months, 45% of the mothers and 38% of the fathers had a score of six, the highest possible satisfaction score. At child-age four years, the results were similar with 48% of mothers and 40% of fathers with a score of six.<br>More frequent staff change was associated with less study satisfaction at 15 months, but not at four years.<br>Mothers had higher scores (M = 4.50, 95% CI 4.46,4.55) than fathers(Mean = 4.12, 95% CI 4.07, 4.17) at 15-months $p < 0.001$. The results were similar at four years (mothers:<br>Mean = 4.59, 95% CI 4.53,4.64; fathers: Mean= 4.18, 95% CI 4.12, 4.25, $p < 0.001$).<br>In the subgroup of parents who completed the 15-month and four-year satisfaction measure,, parent satisfaction scores remained high over time (mothers: 15-months Mean = 4.68, 95% CI 4.62, 4.73 and four-years Mean = 4.60, 95% CI 4.55, 4.66; fathers: 15-months Mean = 4.25, 95% CI 4.19,<br>4.31 and four-years Mean= 4.20, 95% CI 4.13, 4.26).<br>Parent study satisfaction was highest in Sweden and the US, compared to Finland. Parents who had an accurate perception of their child's type 1 diabetes risk and those who believed they can do something to prevent type 1 diabetes were more satisfied.	More educated parents and those with higher depression scores had lower study satisfaction scores
Melin *et al.* 2023 (70)	DiPiS - antibody surveillance	Mothers and Fathers, General Population, genetically predisposed children	Reasons for continuation: Completed more study visits in subsequent 3 years of the study: children from Sweden, mothers with their first child, older mothers, father actively participated, mothers more satisfied with study participation during first year. Of mothers with the highest study satisfaction scores at 15months, 65% completed all study visit between 18 and 48 months compared to 48% mothers least satisfied with the study. Fathers' study satisfaction at 15months also associated with subsequent visit compliance.	Reasons for discontinuation: Completed fewer study visits in subsequent 3 years of study: ethnic minority children, children of mothers who smoked during infancy, mothers had high scores on EPDS and whose mothers expressed higher anxiety about child's T1D risk in first year of TEDDY.

Nicholls *et al.* 2013 (72)	Hypothetical / attitudinal	Parents with a child aged <5 years	Screening preferences (hypothetical): lack of preventative measures was viewed critically. Parents feared potential worry and anxiety from a high risk screening result. Parents thought important considerations for screening were 1) as no prevention, consider targeted screening rather than general population, 2) evidence requirements for the screening test including potential benefits, cost-effectiveness, clinical validity and clinical utility, 3) healthcare system preparedness, particularly need for education and awareness and 4) information usage and third party access.	
Nicholls *et al.* 2016 (73)	Hypothetical / attitudinal	Parents with a child aged <5 years	Screening preferences (hypothetical): 30% of parents supported T1D screening at the start of the workshop and after further information was received, this increased to 41%.	Screening preferences: 28% of parents held a positive attitude towards T1D genetic testing at the start of the workshop compared to 9% at the end. 88% of parents were concerned that T1D screening results would cause extra worry.
Quinn *et al.* 2024 (74)	Hypothetical / attitudinal	Mothers and Fathers, Children, General Population	Screening preferences (hypothetical): Parents emphasised importance of informed consent with clear information to facilitate this. Parents preferred less invasive screening tests and sought support from health care providers and suggested peer support could be beneficial.	Screening preferences: Parents expressed uncertainty about screening and worry about potential anxiety, overly protective behaviours if child were identified at risk and responsibility to monitor for T1D symptoms.
Scudder *et al.* 2024 (75)	T1Early - antibody screening	Mothers, General Population	Reasons for participation: 1) being prepared, time to mentally prepare, gather information and plan ahead was preferable to having to deal with shock of sudden diagnosis, 2) Ruling something out and feeling reassured - participants described wanting to know the outcome, stating having the results provides reassurance.<br><br>Screening experience: 1) Parents described mixed experience of their child's response to the process, described ease of the process, 2) child had recovered after 5-10 minutes, 2) some parents were surprised how well their child reacted and that their child had not mentioned it afterwards, 3) one parent said they had been worried the screening process might give their child lasting fear of going to the doctors but was reassured that their child seemed keen to attend afterwards, 4) in general, parents recalled child's excitement over the stickers, plaster or treat received after their visit, 5) overall parents recalled satisfaction with the process feeling benefits outweighed potential upset. Parents were glad they had taken part and although the experience caused their child distress, overall they felt positive about the experience and related that they would do it again and recommend it to others. All parents stated they would want their child to take part in T1D screening if asked again. 6) Liked it linked to the vaccination programme for the following reasons: busy lives, lack of transport and having to take child out of nursery as reasons why they preferred screening alongside routine health visit, reflected would have been less likely to agree if screening offered separately or in a different location.	Screening experience: 1) negative experiences included having to hold their child securely and the stress caused to the child, although most parents said the child was primarily upset by the vaccinations carried out first, 2) Some parents recalled their child mentioning their finger or arm hurting, 3) parents were shocked at the amount of blood required, and how much pressure the nurse needed to apply, also concern over length of time to collect the blood volume, parents recounted feeling distressed and wishing the nurse would finish and feeling sad on behalf of their child.
Simonen *et al.* 2006 (34)	DPP - newborn genetic screening (cord blood) and antibody surveillance	Mothers and Fathers, General Population	Reasons for participation: 92% high risk Mothers and 95% of high risk Fathers, 96% of low risk Mothers and 95% of low risk Fathers thought it was good to know about the infant's T1D risk.<br><br>Screening experience: Satisfaction was significantly higher in low risk parents than high risk parents: 88% high risk Mothers and 89% high risk Fathers, 98% low risk Mothers and 96% low risk Fathers.	

			Reasons for continuation: 96% of low risk Mothers and Fathers said they would have continued in the study if infant found to be high risk.	
Sims *et al.* 2019 (76)	Trialnet - Antibody surveillance	Parent of antibody positive child	Returned for antibody confirmation: child aged <12 years - 83.8% returned and 16.2% did not, child aged 12-17 years - 80.5% returned and 19.5% did not.<br><br>Returned for OGTT: child aged <12 years - 71.1% returned and 28.9% did not, child aged 12-17 years - 67.7% returned and 32.3% did not.	
Stolt *et al.* 2002 (77)	ABIS - newborn screening (not routinely informed of result)	Mothers, General population	Reasons for participation: 1) Explicit wish to contribute to research was the most common reason for participation (instead of predominantly thinking in terms of what they and their child might gain from participating) (14/15), 2) illness in family e.g. diabetes, allergy, rheumatism, asthma, coeliac, how common these illnesses are in families today 6/15 and 7 Mothers had experience of diabetes/allergy in themselves or close family, 3) wish to detect if their own child will develop diabetes/allergy/rheumatism/coeliac (5/15), 4) can be of general help to my own child (development of medicines, discovering connection between food and illness) 5/15, 5) wish to help other children and families 2/15 - i.e. help (help research, help my own child, help other children, etc.), 6) parents displayed a positive attitude to the ABIS study (21/21), 7) all Mothers thought it was important that they and their family participated in the study.<br><br>Reasons for non-participation: Non participating mothers shared same positive attitude, overall attitude for screening research on children was positive, but had their individual reasons for non-participation: 1) Would have participated if had been asked, 2) not enough information received, otherwise would most likely have said yes 1/5.	Reasons for non-participation: 1) not wanting to put child through frequent blood testing, concern for their child not for themselves 4/5, 2) not wanting child to be part of "experiments" 2/5, 3) feeling of too much work involved/lack of time 2/5, 4) not wanting to know any results (discover an illness) 1/5, 5) A feeling of an anonymous mass project with only diagrams and statistics as results 1/5, 6) bad experiences of projects/healthcare due to chronic disease in family 1/5.
Stolt *et al.* 2003 (78)	ABIS - newborn screening (not routinely informed of result)	Parents, General population	Attitudes to screening: 83% had a positive attitude, 80% felt actively involved and 97% of the respondents regarded this research as important.<br><br>Reasons for participation: most frequently cited was the wish to contribute to research (87.3%), followed by opportunity to help their own or other children (81.2%). 23.8% said they had diseases in their own family which drove study entry. 15.8% of mothers said the main reason for participation was the potential benefit for their child.	
Swartling *et al.* 2008. Parental (79)	ABIS - newborn screening (not routinely informed of result)	Children aged 10-14 years	Reasons for participation: 1) main reason for participation was to aid research and might aid other children, 2) significantly lower belief that their participation would be of potential therapeutic use to their child, 3) 93% had positive attitude towards the ABIS study and its aims.	

Swartling *et al.* 2011 (81)	ABIS - newborn screening (not routinely informed of result)	Parents	Reasons for participation: Sense of altruism: 1) research may lead to potential benefits for others, research was to help people not only children, 2) children with experience of ABIS said they want children to be in research since almost all know someone (child) who is sick or has disease, 3) children want to participate so as to help children so they don't get ill, and those you help can help in others in return, 4) children knew they received a movie ticket for focus group participation, homogenous feeling that reward was sometimes good but not in setting of real research where it was seen as bribery (real research meant finding treatments and medicines), then you might not participate to help others but just for the incentive, children want to participate in research and nothing needs to be given, willingness to help others without personal gain.	
Swartling *et al.* 2014 (80)	ABIS - newborn screening (not routinely informed of result)	Mothers and Fathers, General Population	Reasons for participation: Participation in medical research like ABIS, 1) 25% marked very important (lower than 65-86% rating medical research as important in general), 2) children with earlier experience of ABIS were more positive, 3) Research on why people get sick and research on drugs is very important, 65-86%, girls>boys, 4) trust in researchers emphasised by all children, 97%, girls>boys.<br><br>Screening experience: most reported feeling calm about participation in ABIS.	
Van Esch *et al.* 2010 (82)	Hypothetical / attitudinal	Diabetes patients, partners and relatives	Screening preferences (hypothetical): In pregnancy, mothers sought information about the child's risk of T1D or T2D and heritability information.	
Wentworth *et al.* 2025 (83)	Type1 Screen – FDR testing	Children, FDR	71% of children opted for in-home blood spot selection as the screening test vs 29% who selected venepuncture. Those who did not complete an in-home blood spot were younger and more likely to be male than those who did.	

ESM Table 6 legend – Articles providing positive (green) and negative (red) indicators of acceptability for screening or early detection of paediatric type 1 diabetes. Abbreviations: Aab – autoantibody, T1D – type 1 diabetes, FHx – family history of type 1 diabetes, FDR – first degree relative, DKA – diabetic ketoacidosis, DBS – dried blood spot, GP – general population, OGTT – oral glucose tolerance test.

ESM Table 7 - Summary of findings

	Bennett-Johnson *et al.* 2011 (10)	Sims *et al*. 2021 (2)	Phillip e*t al.* 2024 (11)	Systematic review and mixed methods evidence synthesis - New findings
**Emotional implications**	1. Children and parents experience worry or concern from high risk result.<br>2. Initial high anxiety.<br>3. Anxiety wanes.<br>4. Predictors of anxiety:<br>a. Finland - higher anxiety<br>b. Family history (FHx)<br>c. Low education and ethnic minority<br>d. History depression<br>e. Coping style – self-blame, wishful thinking, avoidance	1. Negative psychological impact for screen positive results.<br>2. Stress wanes overtime.<br>3. Adjustment to diagnosis is favourable with screening and monitoring compared to usual care.<br>4. Screening facilitates access to ongoing education and monitoring.	1. Significant stress from islet autoantibody detection. Responses include shock, grief, guilt, anger, depression and anxiety.<br>2. Mothers experienced more anxiety than fathers.<br>3. Anxiety decreases for parents of children who did not develop antibodies, however anxiety remained high for parents with a multiple antibody positive child.<br>4. Increased anxiety observed in mothers who experienced negative interpersonal life events, post-partum depression, increased risk perception accuracy or parents with a lower educational level.<br>5. In Fr1da, 40% mothers and 20% fathers experienced elevated symptoms of depression following antibody detection compared to 18% of mothers/fathers of an antibody negative child. Depressive symptoms declined over 12 months.<br>6. Monitoring can reduce emotional implications of screening.	1. Previous findings confirmed in general population antibody screening programmes<br>2. Anxiety may persist or recur – supported by qualitative data<br>3. Anxiety following participation in intervention trial<br>4. Predictors of anxiety: multiple versus single Aab positive, extremely high genetic risk, single mothers
**Cognitive implications**	1. Initial accurate risk perception.<br>2. Risk accuracy wanes overtime.<br>3. More accurate risk perception:<br>a. FHx families<br>b. Mothers>fathers<br>4. Less accurate risk perception:<br>a. Lower education, ethnic minority,	Not reported	1. Education to inform about T1D symptoms reduces parenting stress.	1. Previous findings confirmed in general population antibody screening programmes.<br>2. Risk interpretation varied – supported by qualitative data<br>3. Predictors of accurate perception: high risk classification, maternal anxiety.

	maternal depression			
**Behavioural implications**	1. Frequent monitoring behaviours aiming to prevent type 1 diabetes (T1D) – e.g. glucose monitoring, lifestyle changes.	Not reported	1. Parents frequently engage in behaviours aiming to prevent T1D.<br>2. Dietary changes are most frequently reported, particularly in FDR families.	1. Quantified numbers performing behavioural changes.<br>2. Monitoring behaviours more common in FDR than general population families.<br>3. Evidence of restrictive behaviours – supported by qualitative data<br>4. Predictors of behaviour change: older maternal age, higher maternal education, only child, FHx family, higher anxiety of depression score, accurate risk perception, belief risk could be modified, living in US/Germany/Sweden compared to Finland.
**Acceptability**	Parents want to know child's risk	1. Parents value screening programmes which reduce diabetic ketoacidosis risk, offer monitoring and facilitate access to treatment or trials to delay T1D onset.	Not reported	1. Motivations for screening – time to prepare, monitoring, possibility of prevention, T1D in the family.<br>2. Reservations about screening - blood taking, lack of time, not wanting to know risk.<br>3. Screening experience – positive and negative reflections, supported by qualitative data.
**Hypothetical preferences**	Not reported		Not reported	1. Prefer preventative treatment.<br>2. Concerns about insurance and potential emotional and behavioural implications of screening.
**Research ethics**	Not reported	Not reported	Not reported	1. Decision making preferences.<br>2. Results disclosure preferences.<br>3. Data integrity preferences.

**Recommendations relating to implications and acceptability of paediatric type 1 diabetes screening**	1. Need to monitor families' risk perception to ensure successful risk communication.<br>2. Provide psycho-educational support and resources to those in need.<br>3. Need to monitor behaviour changes.	1. Need to understand patient perspectives on monitoring and treatment.<br>2. Improve engagement with traditionally under-represented groups.<br>3. Ensure education, ongoing communication and partnerships with healthcare providers to optimise outreach and ensure continued patient engagement and care.<br>4. Identify optimal screening strategies – antibody versus genetic testing and implications of these approaches.<br>5. Identify optimal strategies for risk communication and to ensure continued engagement.	1. Education tailored to stage, family history of T1D and relative timepoint and delivered by healthcare providers experienced in Pre-T1D.<br>2. Education offered at key life transitions and critical timepoints, i.e. initial diagnosis, follow-ups, and annual reviews.<br>3. Educate on the implications of screening results and benefits of adherence to monitoring.<br>4. Offer multiple modalities for education, including virtual and in-person sessions.<br>5. Urgent need for guidance on psychosocial support.<br>6. Emotional, cognitive and behavioural implications should be assessed and addressed.<br>7. Assess coping, anxiety and depression at each monitoring visit using age-appropriate and validated questionnaires or global measures of psychosocial functioning.<br>8. Consider developmental and family factors in relation to psychosocial needs.<br>9. Aim to integrate psychosocial care into routine visits, adopting a collaborative, person-centred, culturally sensitive approach and delivered by healthcare providers with relevant training.<br>10. Assign a contact person to answer questions, provide age-specific education and act as role models.	1. Need to identify those experiencing adverse implications of screening to facilitate access to early psychosocial support.<br>2. To assess long-term implications of screening.<br>3. To investigate the implications of screening on children and young people.<br>4. Need qualitative studies to explore the implications and acceptability for general population antibody screening programmes.

ESM Table 7 Legend
New findings from this systematic review compared to previous narrative reviews. References: 2, 10, 11. Abbreviations: Aab – autoantibody, T1D – type 1 diabetes, FHx – family history of type 1 diabetes, FDR – first degree relative.
